# The treasure inside barley seeds: microbial diversity and plant beneficial bacteria

**DOI:** 10.1186/s40793-021-00389-8

**Published:** 2021-10-28

**Authors:** Nina Bziuk, Lorrie Maccario, Benjamin Straube, Gwendolin Wehner, Søren J. Sørensen, Adam Schikora, Kornelia Smalla

**Affiliations:** 1 Institute for Epidemiology and Pathogen Diagnostics, Julius Kühn Institute (JKI) – Federal Research Centre for Cultivated Plants, Messeweg 11-12, 38104 Braunschweig, Germany; 2 Section of Microbiology, Copenhagen University, Universitetsparken 15, 2100 Copenhagen, Denmark; 3 Institute for Resistance Research and Stress Tolerance, Julius Kühn Institute (JKI) – Federal Research Centre for Cultivated Plants, Erwin-Baur-Str. 27, 06484 Quedlinburg, Germany

**Keywords:** Endophytes, Seed microbiome, Rhizosphere microbiome, Bioassays, PGPR, *Hordeum vulgare*, Genotypes, Breeding strategies, Beneficial microbes

## Abstract

**Background:**

Bacteria associated with plants can enhance the plants’ growth and resistance against phytopathogens. Today, growers aim to reduce the use of mineral fertilizers and pesticides. Since phytopathogens cause severe yield losses in crop production systems, biological alternatives gain more attention. Plant and also seed endophytes have the potential to influence the plant, especially seed-borne bacteria may express their beneficiary impact at initial plant developmental stages. In the current study, we assessed the endophytic seed microbiome of seven genetically diverse barley accessions by 16S rRNA gene amplicon sequencing and verified the in vitro plant beneficial potential of isolated seed endophytes. Furthermore, we investigated the impact of the barley genotype and its seed microbiome on the rhizosphere microbiome at an early growth stage by 16S rRNA gene amplicon sequencing.

**Results:**

The plant genotype displayed a significant impact on the microbiota in both barley seed and rhizosphere. Consequently, the microbial alpha- and beta-diversity of the endophytic seed microbiome was highly influenced by the genotype. Interestingly, no correlation was observed between the endophytic seed microbiome and the single nucleotide polymorphisms of the seven genotypes. Unclassified members of *Enterobacteriaceae* were by far most dominant. Other abundant genera in the seed microbiome belonged to *Curtobacterium*, *Paenibacillus*, *Pantoea*, *Sanguibacter* and *Saccharibacillus*. Endophytes isolated from barley seeds were affiliated to dominant genera of the core seed microbiome, based on their 16S rRNA gene sequence. Most of these endophytic isolates produced in vitro plant beneficial secondary metabolites known to induce plant resistance.

**Conclusion:**

Although barley accessions representing high genetic diversity displayed a genotype-dependent endophytic seed microbiome, a core seed microbiome with high relative abundances was identified. Endophytic isolates were affiliated to members of the core seed microbiome and many of them showed plant beneficial properties. We propose therefore that new breeding strategies should consider genotypes with high abundance of beneficial microbes.

**Supplementary Information:**

The online version contains supplementary material available at 10.1186/s40793-021-00389-8.

## Background

Seed endophytes gain increased attention due to plant beneficial characteristics that are of great interest for crop protection. Yield losses caused by phytopathogens in agricultural systems are estimated to reach 20–30% worldwide [1]. As the application of pesticides should be reduced in the European Union [2], biological control of phytopathogens, *e.g.* via seed treatments with beneficial bacteria, is an ecologically friendly alternative. Additionally to seed treatments, seed endophytes are expected to serve as potential source of breeding targets [3]. Plant seeds harbor a number of different microbes which were shown to include diverse plant beneficial bacteria [4, 5].

Barley (*Hordeum vulgare*) is an efficient model crop plant due to its worldwide distribution [6] and completely sequenced genomes [7–10]. The yield losses of barley can reach worldwide up to 30%, mostly caused by diseases, pests and weeds [11, 12]. Strategies like defense priming or induced resistance to improve the plants' defense response towards phytopathogens already attracted some research interest [13, 14]. The induction of resistance (induced systemic resistance; ISR) can occur by a wide range of biotic or abiotic agents [15]. Antibiotics like pyocyanin, or siderophores, *N*-acyl homoserin lactones (AHLs), 2,4-diacetylphloroglucinol (2,4-DAPG) and biosurfactants were already identified as ISR elicitors for different plants [16, 17]. Beneficial plant-associated bacteria may also influence the plant immune system, as shown on multiple occasions [15, 18, 19]. In this context, also plant endophytes could play an important role, as observed for *Acidovorax radicis* N35 that was able to prime barley plants by secretion of AHLs [20]. However, not much is known of the ISR potential of innate microbes in barley seeds.

Rahman et al. [5] observed *Paenibacillus*, *Pantoea* and *Pseudomonas* as major seed endophytes in various barley genotypes originating from different geographical sites and harvest years. Some of the obtained isolated endophytes showed plant growth promotion potential in vitro and in vivo [5]. However, the authors did not investigate alpha- or beta-diversity of the microbiome and did not focus on the influence of the genotype [5]. Yang et al. [21] examined the metabolically active part of the barley seed microbiome based on total RNA from activated seeds. Although the authors found that the microbiome in barley seeds of modern commercially available cultivars was influenced by its genotype, they also observed a core microbiome consisting of 21 OTUs belonging to *Paenibacillaceae*, *Enterobacteriaceae* and *Pseudomonadaceae* [21]. Nevertheless, nothing was known about the genetic relatedness between the investigated cultivars and the microbial functions of the seed microbiome.

Induced resistance by chemical elicitors, as well as by bacterial inoculants, was reported to depend on the barley genotype in both field and greenhouse experiments [14, 22, 23]. In a previous study, Wehner et al. [14] identified five barley accessions out of a worldwide set of 224 diverse spring barley accessions (called GENOBAR set [24]) and two reference genotypes that represent the genetic diversity within the GENOBAR set (barley 7’set). The barley 7’set was analyzed for its AHL-priming induction towards *Puccinia hordei* and genotype-dependent differences were revealed [14]. Nevertheless, the role of the associated microbiome was not investigated.

The present study aimed to elucidate whether the plant genetic diversity influences the seed and rhizosphere microbiome of the barley 7’set. We hypothesized that seeds of the barley 7’set harbor a plant genotype-dependent microbiome including beneficial endophytes with potential resistance enhancing capacities. As 16S rRNA gene amplicon sequencing provides only limited information on the functions of the microbiome, we isolated endophytes from barley seeds, determined their 16S rRNA gene sequence and assessed potential plant beneficial activities. Furthermore, we explored the contribution of plant genotype and its seed microbiome to the rhizosphere microbiome of the barley 7’set grown in an agricultural soil.

## Material and methods

### Plant material

For the present study, we were using the barley reference genotypes Golden Promise and Morex, since these are genetically characterized [7, 8], additionally to the five accessions of barley BCC436, BCC768, BCC1415, BCC1598 and HOR7985, representing the genetic diversity within the GENOBAR set [14, 24]. The set of accessions is hereinafter called barley 7’set. Untreated seeds were obtained from all genotypes (Morex, BCC436, BCC1415, BCC768, BCC1598 and HOR7985) that were previously grown under the same growth conditions at the field station of the Julius Kühn Institute in Groß Lüsewitz, Germany. Untreated seeds of Golden Promise were obtained from Simpsons Malt Limited (Berwick-upon-Tweed, United Kingdom).

### Cultivation-independent analysis of the barley seed microbiome

DNA for the analysis of the endophytic seed microbiome was extracted from four replicates per genotype, each consisting of six seeds. Barley seeds were first surface-sterilized modified after [25] using one time 2% sodium hypochlorite and no ethanol and swelling. Seeds were dried over night at room temperature. Surface-sterilized seeds were ground in a sterile mortar using liquid nitrogen. DNA from the seed powder was extracted as described in [26].

### Determination of the rhizosphere microbiome composition of the barley 7’set

The barley 7’set (four replicates each) was planted under greenhouse conditions into two soil variants of a long-term field experiment located in Bernburg, Germany, where two contrasting tillage practices were previously applied: conventional mouldboard plough (MP, 20–30 cm depth) and conservation cultivator tillage (CT; 12–15 cm depth), both additionally fertilized with 220 kg/ha N and fungicide application [27]. The barley 7’set was grown in approx. 100 g soil (one plant per pot) at 18 °C and 16/8 h (day/night) photoperiod. The plants were watered with tap water according to demand. Unplanted pots (four replicates each) served as bulk soil control. Rhizosphere and bulk soil samples were taken at the three-leaf growth stage (BBCH13, [28]) as described in [29]. DNA of rhizosphere and bulk soil samples was extracted using the FastDNA™ Spin Kit for Soil (MP Biomedicals, Eschwege, Germany) according to the manufacturer’s instructions.

### Preparation of 16S rRNA gene amplicon libraries

Amplicon sequencing libraries of seed and rhizosphere samples were prepared using a two-step PCR, targeting the 16S rRNA gene’s V4 region, with primers listed in Additional file 1: Table S1. First PCR was performed using the primers 515F and 806R modified from [30] and flanked with Illumina adapter priming sequences. To limit amplification of plant DNA in seed samples, additional mitochondrial and chloroplast blocking primers based on a C3 Spacer at the 3’-end were used, as it was previously proposed [31, 32] and adopted from [30] with additional reverse primer 802R. The PCR reaction was performed as follows: the reaction volume of 25 µL included 0.5 u Phusion HF DNA Polymerase, 1 × Phusion HF Buffer, 200 µM of each dNTP, 3% dimethyl sulfoxide (DMSO), 0.1 mg/mL bovine serum albumin (BSA), 0.25 µM of each 16S rRNA gene primer, 2.5 µM of each blocking primer and 1 µL of the respective DNA. The thermocycler program was set at 98 °C for 30 s, 30 cycles of 10 s at 98 °C, 15 s at 61 °C, 30 s at 72 °C, and a final elongation step for 5 min at 72 °C. PCR products were verified by agarose gel electrophoresis. One replicate of Golden Promise seed samples was discarded due to a low amount of amplicons. First PCR amplification products were purified using HighPrep PCR clean-up (MagBio Genomics, Gaithersburg, MD, USA) using a 0.65:1 (beads:PCR reaction) volumetric ratio.

A second PCR reaction was performed priming Illumina sequencing adapters and adding sample-specific dual indices (Nextera XT Index Kit v2 Set D, Illumina, San Diego, CA, USA) using PCRBIO HiFi (PCR Biosystems Ltd., London, UK) for 15 amplification cycles. The products of the second PCR were purified with HighPrep PCR Clean Up System, as described for the first PCR. Sample concentrations were normalized using the SequalPrep Normalization Plate (96) Kit (Thermo Fisher Scientific, Waltham, MA, USA), following manufacturer’s instructions. The libraries were pooled and concentrated using DNA Clean and Concentrator-5 Kit (Zymo Research, Irvine, CA, USA). Library pool’s concentration was determined using the Quant-iT High-Sensitivity DNA Assay Kit (Life Technologies, Carlsbad, CA, USA) and diluted to 4 nM. The library was denatured, diluted to 9 pM and sequenced following manufacturer’s instructions, on an Illumina MiSeq platform at the Section of Microbiology, University of Copenhagen (Denmark) using Reagent Kit v3 [2 × 300 cycles] (Illumina, San Diego, CA, USA) and including 1st and 2nd PCR negative controls, as well as a mock control.

### Generation of amplicon sequence variants

Cutadapt v.2.3 [33] was used to remove primer sequences of the first PCR (515F, 802R and 806R), both on the 5’ and the reverse complement on 3’ ends, also discarding read pairs for which none of the forward or reverse primers could be detected. Reads were further processed for error correction, merging and amplicon sequence variants (ASVs) generation using DADA2 version 1.10.0 [34] plugin for QIIME2 [35] with the following parameters: truncL = 270, truncR = 95; trimL = 8, trimR = 8 and maxEE of 2, based on the Qiime2 run quality plot. Each ASV sequence was taxonomically annotated using q2-feature-classifier classify-sklearn module trained with SILVA SSU rel. 132 database [36], trimmed for V4 region only.

The ASV dataset was further cleaned using RStudio (RStudio, Boston, MA, USA) version R3.6.3 with the package phyloseq [37] resulting in removal of 25% of ASVs considered as spurious while retaining more than 85% of the reads. Further, ASVs assigned to unassigned kingdom and phylum (374 ASVs representing 0.28% of the reads in total) were removed, as well as chloroplast sequences (152 ASVs representing 0.66, 0.48 and 36.09% of the reads in bulk soil, rhizosphere and seed samples, respectively) and mitochondria sequences (116 ASVs representing 0.09, 0.16 and 46.40% of the reads in bulk soil, rhizosphere and seed samples, respectively). Decontam [38] was used to remove potential contaminants as determined by the prevalence of ASVs in the negative controls (from first and second PCRs, 11 ASVs representing 0.12% of the reads in real samples). ASVs were also filtered based on 16S rRNA V4 region size, retaining only sequences of a length between 260 and 280 bp after adapters and primers trimming (removing 2 ASVs = 5 reads).

### Amplicon sequencing data analysis

Alpha-diversity indices for the seed and rhizosphere microbiome were calculated based on read count data 100 times randomly subsampled to the lowest number of sequences (seed microbiota: 1475; rhizosphere microbiota: 6203) using RStudio R3.6.3 packages multcomp [39] and vegan [40]. Analysis of variance (ANOVA), permutational analysis of variance (PERMANOVA), non-metric multidimensional scaling (NMDS) and analysis of similarities (ANOSIM) were performed with vegan package and based on read count data. PERMANOVA was conducted based on Bray–Curtis dissimilarity indices (10,000 permutations). Venn diagrams were conducted using the packages VennDiagram [41] and venn [42]. Tukey’s test was performed with SAS 9.4 (SAS Institute, Cary, NC, USA), *p* ≤ 0.05 was assumed as different.

### Correlation between seed microbiome and genetic relatedness

The barley 7’set was analyzed with the barley 9 k iSelect single nucleotide polymorphisms (SNP) chip [43] by SGS—Trait Genetics (Gatersleben, Germany) [14]. Additionally, genotyping by sequencing (GBS) data for the respective genotypes were used [44]. After filtering by 5% minor allele frequency, 12.5% heterozygosity and 10% missing values, 23,418 SNPs were used for the following analyses.

A distance matrix of SNPs from the different genotypes was prepared using adegenet [45] and poppr [46] in RStudio R3.6.3. The distance matrix of the microbiota based on averaged ASVs of each genotype was created with vegan [40] package. A Mantel test based on Spearman’s rank correlation with 10,000 permutations was conducted using geosphere [47].

### Cultivation-dependent isolation of barley seed endophytes

Barley seeds (four replicates per genotype, each consisting of 12 seeds corresponding to circa 0.5 g) were surface-sterilized as described above. Surface-sterilized seeds were ground in a sterile mortar under sterile conditions and the seed paste was transferred to a 50 mL tube. 4.5 mL sterile double-distilled water was added and a dilution series of up to 10^−3^ was plated on R2A supplemented with 100 µg/mL cycloheximide to reduce fungal growth. The plates were incubated at 28 °C and colony forming units (CFUs) were determined after 24 h, 48 h and seven days. Seed paste of the genotype Golden Promise was additionally incubated as liquid culture with 4.5 mL buffered peptone water (BPW) at 28 °C over night. Afterwards, a dilution series was plated up to 10^−6^ on R2A supplemented with 100 µg/mL cycloheximide and incubated at 28 °C for 24 h.

### Identification of barley seed endophytes

Ten bacterial isolates were chosen randomly for each plant genotype and were further cultivated on R2A. For BCC768, only seven bacterial colonies were available. Genomic DNA of bacterial isolates was extracted using the Genomic DNA Extraction Kit (Qiagen, Hilden, Germany) and the Silica Bead DNA Gel Extraction Kit (Thermo Fisher Scientific, Waltham, MA, USA). BOX-PCR fingerprints were generated for all endophytic isolates [48]. Further analysis of the endophytes based on a sample selection, which depended on the respective BOX fingerprint: for each identical BOX fingerprint, one representative per barley genotype was chosen and the 16S rRNA gene PCR [49] product was sequenced (Macrogen Europe B.V., Amsterdam, Netherlands). The resulting sequences were trimmed and assembled using CLC MainWorkbench 20.0.3 (Qiagen, Aarhus, Denmark). The obtained consensus sequence was blasted to the NCBI database (NCBI, Bethesda, MD, USA). Best NCBI blast hits, endophytic isolate consensus sequences, as well as ASVs from unclassified *Enterobacteriaceae* were truncated to the common region (V4) using Qiime2 feature-classifier extract-reads and Cutadapt [33]. Truncated sequences were aligned using MAFFT [50] on the local Galaxy [51] server at Julius Kühn Institute in Braunschweig, Germany. A phylogenetic tree based on distance matrices of *Enterobacteriaceae*-related ASVs, consensus sequences of *Enterobacteriaceae*-related isolates and NCBI reference sequences was conducted in RStudio using the packages seqinr [52], phangorn [53] and ape [54]. The visualization of the phylogenetic tree was performed with iTOL [55].

Isolate consensus sequences were compared to ASV sequences obtained by 16S rRNA gene amplicon sequencing (see also Table 3) using NCBI BLAST + blastn megablast tool [56, 57] on the local Galaxy server.

### Screening of endophytic isolates for potential plant beneficial activities

Each isolate obtained from seeds of the barley 7’set was tested for potential plant beneficial activities and resistance enhancing capacities. Protease, β-1,3-glucanase and cellulase activity was tested according to [58]. Chitinolytic activity was determined after [59]. Furthermore, the isolates were tested for phosphate solubilization [60], ACC deaminase activity and indole-3-acetic acid (IAA) production [61], as well as the secretion of siderophores [62]. The presence of AHLs were determined in a cross-streak assay using *Chromobacterium violaceum* cv026 for short chain detection of C4-C8 AHLs and *C. violaceum* VIR07 for long chain detection of C10-C16 AHLs [63].

## Results

### Microbial diversity of barley seeds is influenced by the genotype

To test our hypothesis that the genetic diversity of the barley 7’set influences its seed microbiome, we employed sequencing of 16S rRNA gene amplicons from DNA extracted from powder of surface-sterilized seeds. A total of 208,241 reads were obtained and assembled to 475 ASVs. The median read number was 7807 reads per sample. The rarefaction curves (Additional file 1: Fig. S1) showed that the sequence library size was sufficient to cover the microbial diversity in each sample except for one sample of BCC1415 which was not considered in the evaluation due to low amount of reads.

**Fig. 1:**
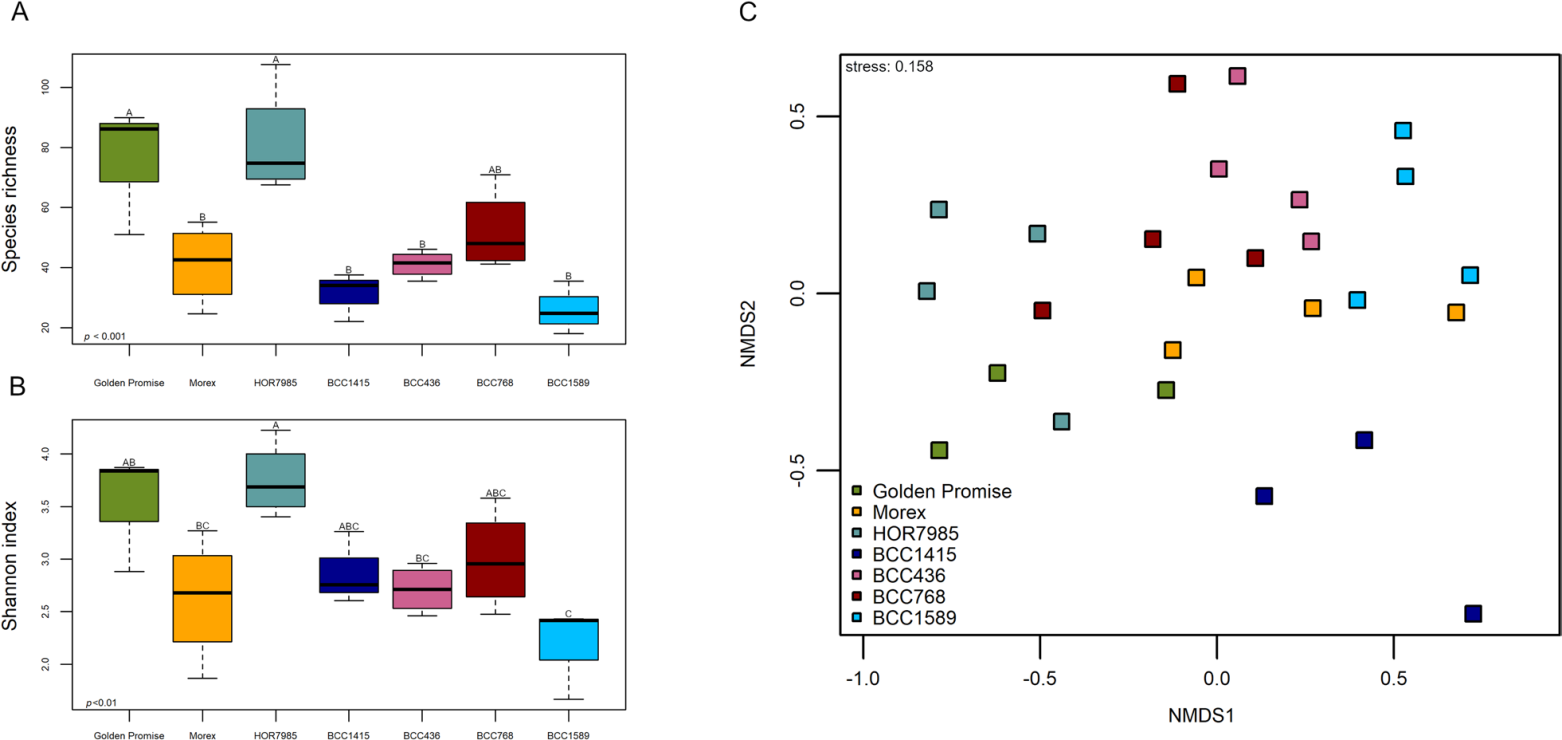
Microbial diversity of the endophytic seed microbiome varied between seven different barley genotypes. Golden Promise and Morex were chosen as reference genotypes. HOR7985, BCC1415, BCC436, BCC768 and BCC1589 represent the genetic diversity within a 224 spring barley accession set [14, 24]. The microbial alpha-diversity indices Species richness (**a**) and Shannon diversity index (**b**) varied depending on the plant genotype (ANOVA for Species richness *p* ≤ 0.001 and Shannon index *p* ≤ 0.01). Values of alpha-diversity indices including statistics can be seen in Additional file 1: Table S2. Beta-diversity of the endophytic seed microbiome was visualized by NMDS (**c**). The microbiome composition based on Bray–Curtis community dissimilarities (ASVs obtained from 16S rRNA gene amplicon sequencing) and was assessed from DNA of surface-sterilized barley seeds of seven different genotypes. ANOSIM verified significant differences between the genotypes (*p* ≤ 0.001)

The influence of the genotype on the endophytic seed microbiome was revealed by one-way ANOVA of the alpha-diversity indices Species richness (*p* ≤ 0.001) and Shannon index (*p* ≤ 0.01). There was only low effect on Pielou’s evenness (*p* ≤ 0.05). Excitingly, Golden Promise and HOR7985 showed the highest microbial alpha-diversity and BCC1589 displayed the lowest microbial alpha-diversity (Fig. 1a, b; Additional file 1: Table S2). However, a pairwise comparison of alpha diversity indices was only significant for few genotypes due to high variability among replicates of the same cultivar.

In order to visualize the beta-diversity of the endophytic seed microbiome, we performed an NMDS (Fig. 1c) revealing a significant grouping of samples belonging to the different genotypes (ANOSIM *p* ≤ 0.001). The genotype BCC1415 showed a clear distinct grouping apart from all other genotypes. Within the closer clustering genotypes, clear patterns emerged for e.g. Golden Promise and HOR7985 compared to BCC1589, whereas BCC436, BCC768 and Morex grouped together.

The significant influence of the genotype on the microbiome composition in barley seeds was supported by PERMANOVA (*R*^2^ = 0.39; *p* ≤ 0.001; Table 1). Testing every genotype in a single analysis against each other disclosed a clearer pattern (Table 1). BCC1415 which showed a separate clustering apart from the other genotypes in the NMDS, was significantly different from all other genotypes, except for Golden Promise. Other significant differences were observed for Golden Promise versus HOR7985, BCC436 and BCC768 and for HOR7985 versus BCC1589 and Morex. To exclude a potential influence of the harvest side, a PERMANOVA without samples of Golden Promise was performed. Although all genotypes were grown at the same field site and under the same growth conditions, their seed microbiome was still significantly influenced by the genotype (PERMANOVA *R*^2^ = 0.37; *p* ≤ 0.01; for NMDS see Additional file 1: Fig. S2).

**Table 1:** PERMANOVA of barley seed microbiome

PERMANOVA	*R*^2^	*p* ≤
Genotype	0.39	0.001
Golden Promise versus Morex	0.28	0.14
Golden Promise versus HOR7985	0.35	0.05
Golden Promise versus BCC1415	0.48	0.1
Golden Promise versus BCC436	0.29	0.05
Golden Promise versus BCC768	0.26	0.05
Golden Pomise versus BCC1589	0.28	0.14
Morex versus HOR7985	0.28	0.05
Morex versus BCC1415	0.43	0.05
Morex versus BCC436	0.08	0.80
Morex versus BCC768	0.18	0.23
Morex versus BCC1589	0.12	0.39
HOR7985 versus BCC1415	0.40	0.05
HOR7985 versus BCC436	0.28	0.06
HOR7985 versus BCC768	0.19	0.14
HOR7985 versus BCC1589	0.34	0.05
BCC1415 versus BCC436	0.39	0.05
BCC1415 versus BCC768	0.32	0.05
BCC1415 versus BCC1589	0.42	0.05
BCC436 versus BCC768	0.10	0.80
BCC436 versus BCC1589	0.12	0.61
BCC768 versus BCC1589	0.14	0.46

Although the barley endophytic seed microbiome was found to be influenced by the genotype for both microbial alpha- and beta-diversity supporting our hypothesis of different microbial communities of the seven genetically diverse barley accessions, no correlation between microbial ASVs and the genetic variation in SNPs of all seven barley genotypes was observed with *r* =  − 0.04 and *p* = 0.41.

### Taxonomic composition of the seed microbiome differs among barley genotypes

**Fig. 2:**
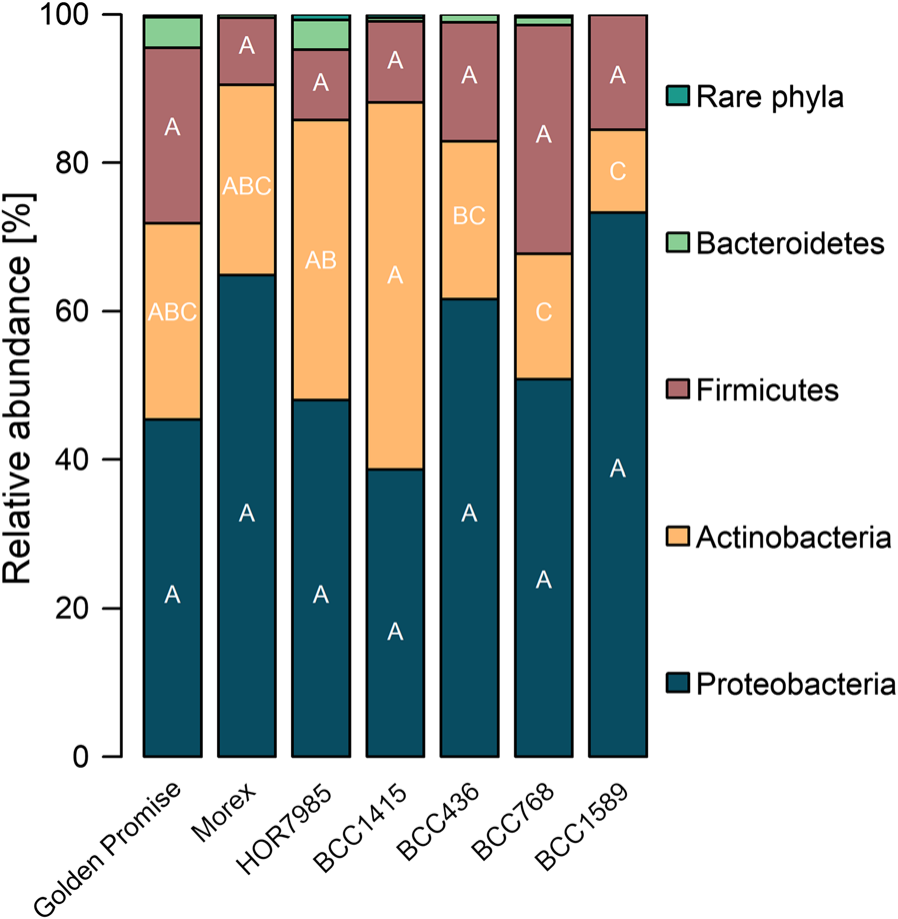
Proteobacteria, Actinobacteria and Firmicutes were dominant phyla in the barley seed microbiome of all genotypes. Four replicates for each genotype, except for Golden Promise (three replicates), were averaged. Values and statistics can be seen in Additional file 1: Table S3

In order to verify our hypothesis that host plant genetic background can influence the composition of the endophytic seed microbiome, we analyzed the taxonomic community composition of the barley seed microbiome. Proteobacteria, Actinobacteria and Firmicutes were revealed as the dominant phyla in the seed microbiome (Fig. 2; Additional file 1: Table S3). Several significant differences between the genotypes were observed for Actinobacteria and the less abundant Bacteroidetes, although a certain variability was displayed by the different replicates of each genotype. For instance, BCC1415 showed a higher relative abundance of Actinobacteria compared to BCC1589 (49.5% vs. 11.2%).

*Enterobacteriaceae*-related taxa were by far the most abundant genera in all genotypes (up to 57.2%; Table 2). We assigned all observed 13 unclassified *Enterobacteriaceae* ASVs to taxonomic groups using the NCBI database to better understand the role of these highly abundant *Enterobacteriaceae* in barley seeds. The two ASV sequences with the highest relative abundance (approx. 66%) were taxonomically affiliated to *Pantoea agglomerans* (99.64–100% sequence identity), whereas the other ASVs revealed a taxonomic affiliation for *Enterobacter* (100% sequence identity) or uncultured *Enterobacteriaceae.*

**Table 2:** Twenty most abundant genera in the barley seed microbiome of the barley 7’set

Phylum/class	Family	Genus	Golden Promise	Morex	HOR7985	BCC1415	BCC436	BCC768	BCC1589
Gammaproteobacteria	*Enterobacteriaceae*	Uncl. *Enterobacteriaceae*	18.39b	50.62ab	22.54b	21.26b	45.55ab	36.73ab	57.20a
Actinobacteria	*Microbacteriaceae*	Curtobacterium*	6.29a	14.14a	9.84a	10.90a	12.71a	5.80a	9.72a
Firmicutes	*Paenibacillaceae*	Paenibacillus*	21.65a	8.59a	1.69a	0.00a	4.02a	3.96a	14.25a
Gammaproteobacteria	*Enterobacteriaceae*	Pantoea*	4.49a	8.12a	2.82a	5.84a	7.76a	5.83a	10.58a
Actinobacteria	*Microbacteriaceae*	Uncl. Microbacteriaceae*	9.20b	4.93b	5.44b	23.61a	4.51b	2.84b	0.44b
Firmicutes	*Paenibacillaceae*	Saccharibacillus*	1.54a	0.46a	3.78a	7.43a	4.41a	10.33a	1.25a
Actinobacteria	*Sanguibacteraceae*	Sanguibacter*	2.11b	2.65b	13.14a	2.06b	1.71b	4.35b	0.31b
Gammaproteobacteria	*Pseudomonadaceae*	*Pseudomonas*	2.47a	1.69a	4.40a	3.39a	6.80a	1.25a	4.17a
Firmicutes	*Bacillaceae*	*Bacillus*	0.48a	0.00a	1.28a	3.48a	0.01a	13.07a	0.00a
Alphaproteobacteria	*Rhizobiaceae*	*Rhizobium*	6.54a	0.72b	6.98a	0.00b	0.37b	2.12b	0.16b
Actinobacteria	*Sanguibacteraceae*	Uncl. *Sanguibacteraceae*	0.94b	1.07b	5.96a	1.41b	1.06b	1.49b	0.26b
Firmicutes	*Family XII*	*Exiguobacterium*	0.00a	0.00a	0.37a	0.00a	7.51a	2.88a	0.00a
Gammaproteobacteria	*Xanthomonadaceae*	Stenotrophomonas*	1.88ab	0.30b	5.01a	0.49ab	0.14b	2.45ab	0.26b
Alphaproteobacteria	*Sphingomonadaceae*	*Sphingomonas*	2.79a	0.73ab	1.51ab	1.46ab	0.35ab	0.79ab	0.00b
Betaproteobacteria	*Burkholderiaceae*	Uncl.* Burkholderiaceae*	3.69a	0.35b	2.22ab	0.20b	0.34b	0.62b	0.04b
Actinobacteria	*Microbacteriaceae*	*Pseudoclavibacter*	0.76b	0.00b	0.17b	7.25a	0.00b	0.00b	0.00b
Actinobacteria	*Nocardiaceae*	*Rhodococcus*	1.90a	0.83a	1.13a	2.40a	0.33a	0.28a	0.08a
Actinobacteria	*Microbacteriaceae*	*Plantibacter*	3.09a	1.27a	0.41a	1.19a	0.08a	0.25a	0.13a
Bacteroidetes	*Weeksellaceae*	*Chryseobacterium*	2.12a	0.13b	1.50ab	0.00b	0.89ab	0.50ab	0.00b
Bacteroidetes	*Sphingobacteriaceae*	*Pedobacter*	1.64ab	0.23bc	1.69a	0.00bc	0.13bc	0.48abc	0.00c

Letters indicate significant differences (Tukey's test *p* ≤ 0.05). Stars indicate genera that were also isolated from seeds

Besides the unclassified *Enterobacteriaceae* and *Pantoea*, highly abundant genera for all barley genotypes were *Curtobacterium*, *Paenibacillus*, *Saccharibacillus* and *Pseudomonas* with similar relative abundances. Excitingly, the relative abundances of some other genera differed between the genotypes. For instance, *Sanguibacter* was about six times more abundant in HOR7985 compared to the other genotypes. Further genera with varying relative abundance were *Rhizobium, Stenotrophomonas, Sphingomonas, Chryseobacterium* and *Pedobacter* (Table 2).

**Fig. 3:**
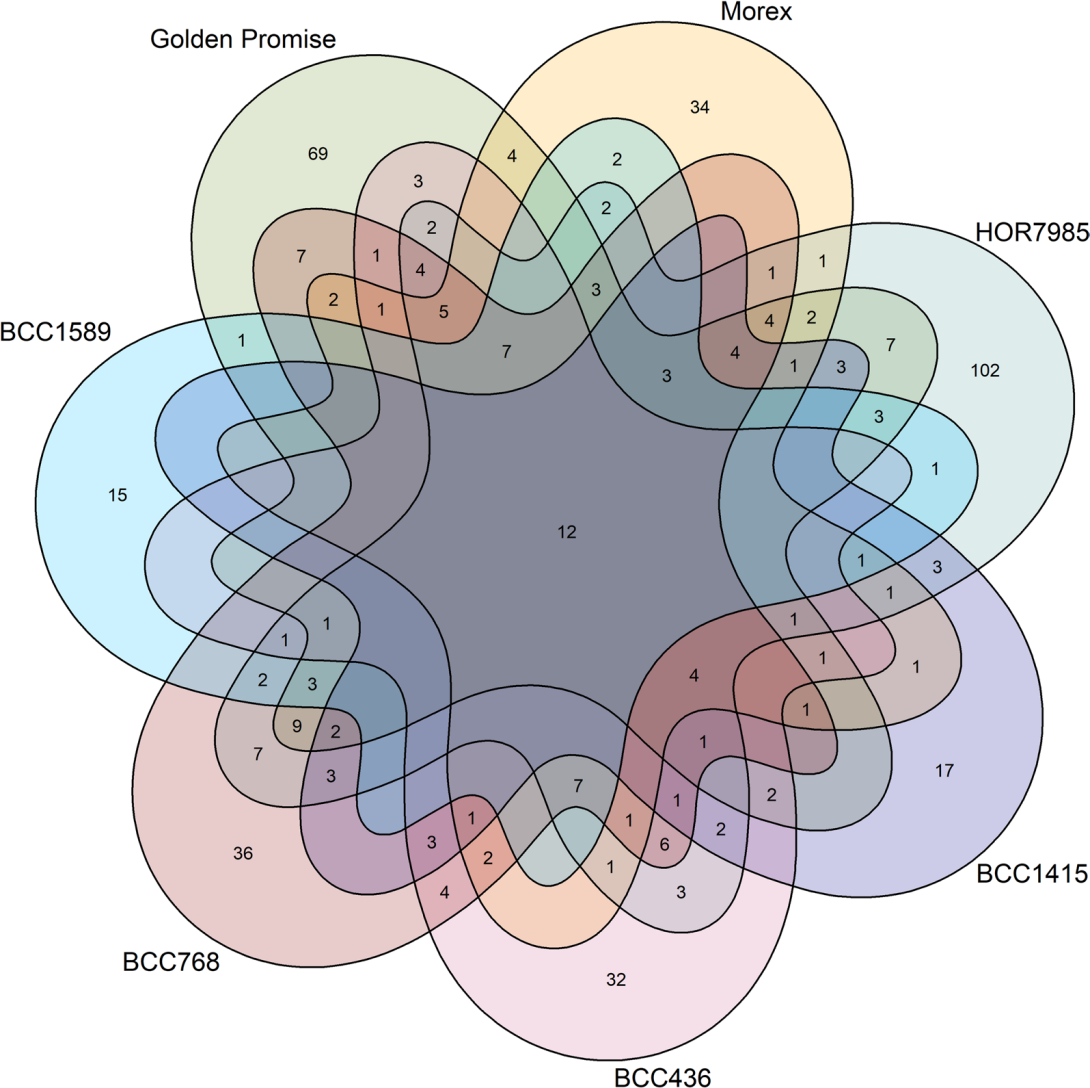
Barley genotypes harbored unique amplicon sequence variants (ASVs), however, they shared a great core microbiome. The numbers of genotype-specific ASVs are higher (25–46%) compared to shared ASVs, however, the shared ASVs made 44% of the reads. The 12 ASVs shared by all genotypes were taxonomically affiliated to *Curtobacterium*, *Pantoea*, *Pseudomonas*, *Sanguibacter*, *Ralstonia*, *Enterobacteriaceae, Sanguibacteraceae* and *Microbacteriaceae*

In total, 475 ASVs belonging to 78 genera were found in the barley seed microbiome. Out of those ASVs, 12 were shared by all genotypes (Fig. 3) and taxonomically affiliated to *Enterobacteriaceae*, *Microbacteriaceae, Sanguibacteriaceae, Curtobacterium*, *Pantoea*, *Pseudomonas*, *Sanguibacter* and *Ralstonia*, and, with about 44% of the reads represented the most abundant genera of all genotypes (Table 2). Every genotype had also 19–45% unique ASVs.

Taken together, the taxonomic composition of the seed microbiome was influenced by the barley genotype. The most abundant genera were shared by the different genotypes indicating a core microbiome, but their relative abundances often differed among the genotypes. Moreover, unique taxa were detected for each genotype as well.

### Prevalence of isolated endophytic bacteria in the barley seed microbiome

In order to provide insights into the functions of the endophytic seed microbiome, we aimed to assign functions to core members of the barley seed microbiome. Therefore, we isolated endophytic bacteria from surface-sterilized seeds of each genotype of the barley 7’set on R2A. The number of CFUs depended on the genotype (Additional file 1: Fig. S2) and ranged from 2*10^2^ for BCC1415 to 4*10^4^ for BCC1589 CFU per g seed. From BCC768 seeds only seven, and from Morex seeds only 10 isolates were obtained. Thus, the colonies picked for further characterization originated from different dilutions.

**Fig. 4:**
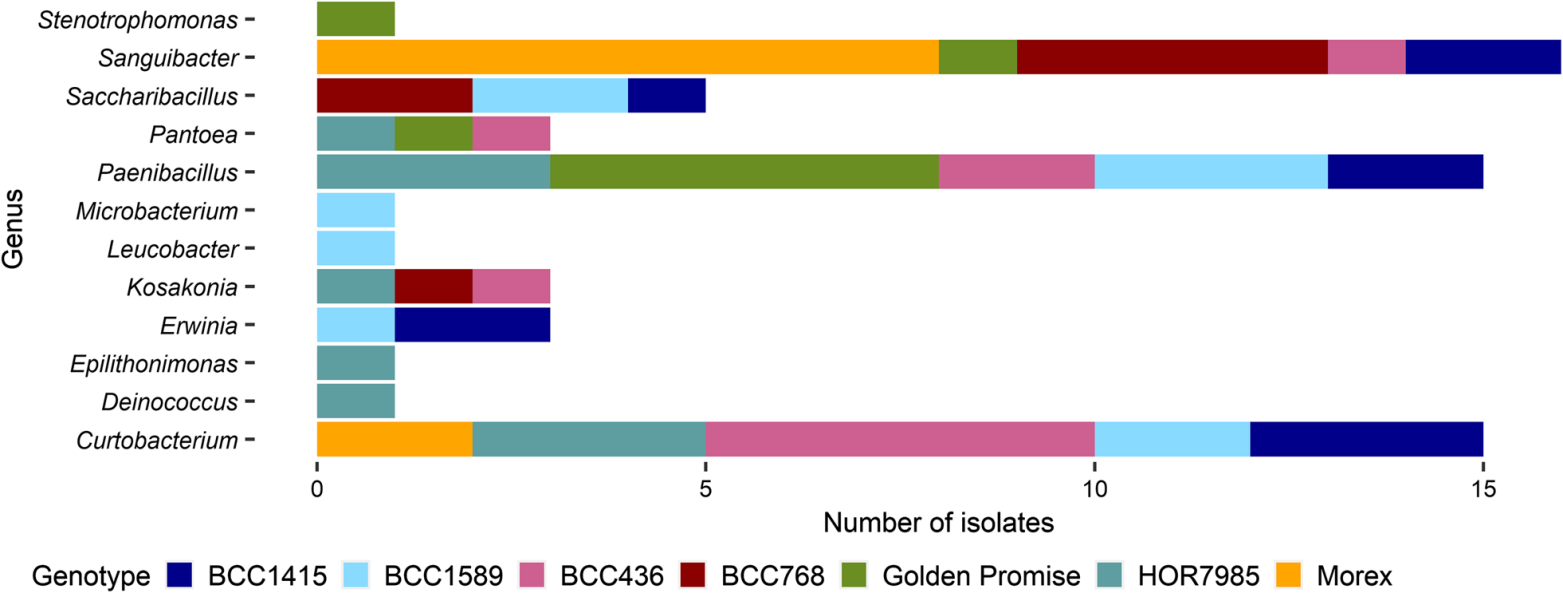
Isolated endophytes from barley seeds of seven different genotypes. Colors represent the different barley genotypes. Taxonomic affiliation of barley seed endophytes was determined by alignment of the 16S rRNA gene amplicon consensus sequence to the NCBI database. The first hit was used for affiliation

The isolated endophytes were grouped according to their BOX fingerprint and a partial sequence of the 16S rRNA gene of one representative was blasted to NCBI database for determination of taxonomic affiliation (Table 3). *Paenibacillus* (21%), *Curtobacterium* (22%) and *Sanguibacter* (24%) were isolated from five of seven genotypes (Fig. 4; Table 3). Additionally, *Pantoea*, *Kosakonia* and *Saccharibacillus* were detected in three of seven genotypes. Other isolates were less frequently obtained. *Curtobacterium* isolates revealed diverse BOX fingerprints independently of the plant genotype from which they were isolated (Additional file 1: Fig. S3). Interestingly, *Paenibacillus* isolates revealed BOX fingerprints differing depending on the genotype (Additional file 1: Fig. S4).

**Table 3:** Functional profile of isolated endophytes from barley seed

Isolate BOX fingerprint (number of strains)	Species (% identity according to NCBI)	% identity to ASV	Protease	β-1,3-glucanase	Cellulase	Chitinase	Phosphatase	ACC deaminase	Siderophore	AHL long chain	AHL short chain	IAA
Morex-5	*Curtobacterium flaccumfaciens* pv. *flaccumfaciens* strain P990 (99.24%)	ASV_2714 (100%)	+	+	+	−	−	−	−	−	−	+
Morex-7	*Curtobacterium flaccumfaciens* pv. *flaccumfaciens* strain P990 (98.81%)	ASV_2714 (99.63%)	+	+	+	−	−	−	−	−	−	(+)
HOR7985-2	*Curtobacterium flaccumfaciens* strain VR38 (97.21)	ASV_2714 (98.90%)	+	(+)	−	−	−	−	−	−	−	+
HOR7985-4	*Curtobacterium* sp. Strain R209Ag (96.11)	ASV_2714 (94.16%)	+	+	+	−	−	−	−	−	−	+
HOR7985-6	*Curtobacterium* sp. 5121 (97.59%)	ASV_2714 (98.90%)	+	+	+	−	−	−	−	−	−	+
BCC1415-3 (2)	*Curtobacterium flaccumfaciens* strain EB337 (98.76%)	ASV_2714 (99.27%)	+	+	+	−	−	−	−	−	−	+
BCC1415-4	*Curtobacterium herbarum* strain EB348 (98.07%)	ASV_2714 (99.27%)	+	−	−	−	−	−	−	−	−	−
BCC436-1 (2)	*Curtobacterium flaccumfaciens* strain EB337 (99.01%)	ASV_2714 (98.91%)	+	(+)/+	+	−	−	−	−	−	−	+
BCC436-3	*Curtobacterium flaccumfaciens* pv. *flaccumfaciens* strain P990 (98.16%)	ASV_2714 (98.55%)	+	+	+	−	−	−	−	−	−	+
BCC436-7	*Curtobacterium flaccumfaciens* strain EB337 (98.47%)	ASV_2714 (99.27%)	+	(+)	+	−	−	−	−	−	−	+
BCC436-10	*Curtobacterium flaccumfaciens* strain EB337 (97.93%)	ASV_2714 (99.27%)	+	−	+	−	−	−	−	−	−	+
BCC1589-10	*Curtobacterium flaccumfaciens* strain EB337 (98.47%)	ASV_2714 (99.27%)	+	++	+	−	−	−	−	−	−	+
BCC1589-3	*Curtobacterium flaccumfaciens* strain EB337 (98.70%)	ASV_2714 (99.27%)	+	(+)	+	−	−	−	−	−	−	+
HOR7985-12	*Deinococcus radiopugnans* strain SEFSRH1 (98.77)	ASV_12237 (83.39%)	−	−	−	−	+	+	−	−	−	+++
HOR7985-1	*Epilithonimonas* sp. ARS123b-11 (97.93%)	ASV_5185 (99.62%)	−	−	−	−	−	−	−	−	−	+++
BCC1415-5	*Erwinia* sp. strain KUDC3013 (97.47%)	ASV_13403 (98.21%)	−	−	−	−	+	(+)	+	−	−	+++
BCC1415-10	*Erwinia* sp. strain KUDC3014 (98.16%)	ASV_13403 (97.86%)	−	−	−	−	+	−	+	+	−	+++
BCC1589-1	*Erwinia* sp. strain KUDC3013 (96.86%)	ASV_13403 (98.56%)	−	−	−	−	+	(+)	+	+	−	+++
HOR7985-3	*Kosakonia* sp. Strain P52 (96.09%)	ASV_13391 (97.43%)	−	−	−	−	+	−	+	−	−	++
BCC436-2	*Kosakonia cowanii* strain Esp (97.20%)	ASV_13391 (98.19%)	−	−	−	−	+	−	+	−	−	++
BCC768-4	*Kosakonia cowanii* strain IHB B 17,550 (96.45%)	ASV_13391 (99.27%)	−	−	−	−	+	−	+	−	−	++
BCC1589-7	*Leucobacter* sp. strain BIS1127 (97.93%)	ASV_2724 (98.91%)	−	−	−	−	−	−	−	−	−	++
BCC1589-8	*Microbacterium* sp. VA8728_00 (99.05%)	ASV_2816 (99.62%)	−	++	−	(+)	−	−	−	−	−	++
Golden Promise-5 (5)	*Paenibacillus* sp. Strain UASWS1731 (99.27%)	ASV_7346 (99.63%)	+	+	+	+	−	−	−	−	−	++
HOR7985-7 (3)	*Paenibacillus* sp. Strain P_s_AC5A (97.89)	ASV_7347 (99.23%)*	+	+	+	±	−	(+)/−	−	−	−	++
BCC1415-1	*Paenibacillus* sp. TI45-13ar (97.89%)	ASV_7346 (100%)*	+	++	+	(+)	−	−	−	−	−	+
BCC1415-7	*Paenibacillus* sp. TI45-13ar (97.84%)	ASV_7346 (100%)*	+	++	+	−	−	−	−	−	−	+
BCC436-9 (2)	*Paenibacillus* sp. TI45-13ar (97.65%)	ASV_7346 (100%)*	+	++	+	±	−	−	−	−	−	++
BCC1589-2	*Paenibacillus* sp. TI45-13ar (98.40%)	ASV_7346 (99.57%)	+	++	+	+	−	−	−	−	−	++
BCC1589-9 (2)	*Paenibacillus* sp. TI45-13ar (99.01%)	ASV_7346 (99.63%)	+	++	+	+	−	−	−	−	−	++
Golden Promise-9	*Pantoea agglomerans* strain SSH (98.56%)	ASV_13403 (100%)*	−	−	−	−	+	+	+	−	(+)	+++
HOR7985-9	*Pantoea* sp. Strain Awest_I_3B (96.22%)	ASV_13403 (97.46%)	−	−	−	−	+	+	+	−	+	+++
BCC436-8	*Pantoea* sp. CH-N10 (98.01%)	ASV_13403 (98.92%)	−	−	−	−	+	−	+	+	−	+++
BCC1415-2	*Saccharibacillus* sp. WB 17 (96.22%)	ASV_7417 (99.26%)	−	−	+	−	−	−	−	−	−	−
BCC768-2	*Saccharibacillus deserti* strain WLJ055 (97.01%)	ASV_7417 (99.63%)	−	+	(+)	−	−	+	−	−	−	−
BCC768-5	*Saccharibacillus deserti* strain WLJ055 (97.09%)	ASV_7426 (96.08%)*	−	+	+	−	−	+	(+)	−	−	++
BCC1589-4	*Saccharibacillus* sp. WB 17 (95.88%)	ASV_7444 (95.37%)	−	(+)	+	−	−	−	−	−	−	−
BCC1589-5	*Saccharibacillus brassicae* strain ATSA2 (95.17%)	ASV_7421 (98.70%)	−	+	+	−	−	(+)	−	−	−	−
Golden Promise-4	*Sanguibacter* sp. Strain NN06 (99.42%)	ASV_2905 (99.63%)	−	(+)	−	−	−	(+)	−	−	−	−
Morex-2 (8)	*Sanguibacter inulinus* strain KAR59 (97.39%)	ASV_2905 (97.14%)	−	−	±	−	−	(+)/−	−	−	−	−
BCC1415-9 (2)	*Sanguibacter* sp. b222 (98.00%)	ASV_2905 (98.55%)	−	(+)/−	−	(+)/−	−	−	−	−	−	−
BCC436-4	*Sanguibacter inulinus* strain KAR59 (96.39%)	ASV_2905 (97.46%)	−	−	−	−	−	−	−	−	−	−
BCC768-1	*Sanguibacter inulinus* strain 12H (98.74%)	ASV_2905 (99.27%)	−	+	−	−	−	+	−	−	−	−
BCC768-3 (2)	*Sanguibacter inulinus* strain 12H (98.11%)	ASV_2905 (98.53%)	−	+	−	−	−	−	−	−	−	−
BCC768-7	*Sanguibacter* sp. Strain NN06 (98.00%)	ASV_2905 (99.63%)	−	+	−	−	−	+	−	−	−	−
Golden Promise-1 (3)	*Stenotrophomonas* sp. Es35 (96.97%)	ASV_13942 (99.63%)	+	−	−	−	−	−	(+)	−	−	+

The isolates are named after the genotype they derived from and are sorted according to the BOX fingerprint

The 16S rRNA gene sequences of the endophytic isolates were further compared with ASV sequences by multiple alignment. Most of the isolate consensus sequences were well presented in ASV sequences.

**Fig. 5:**
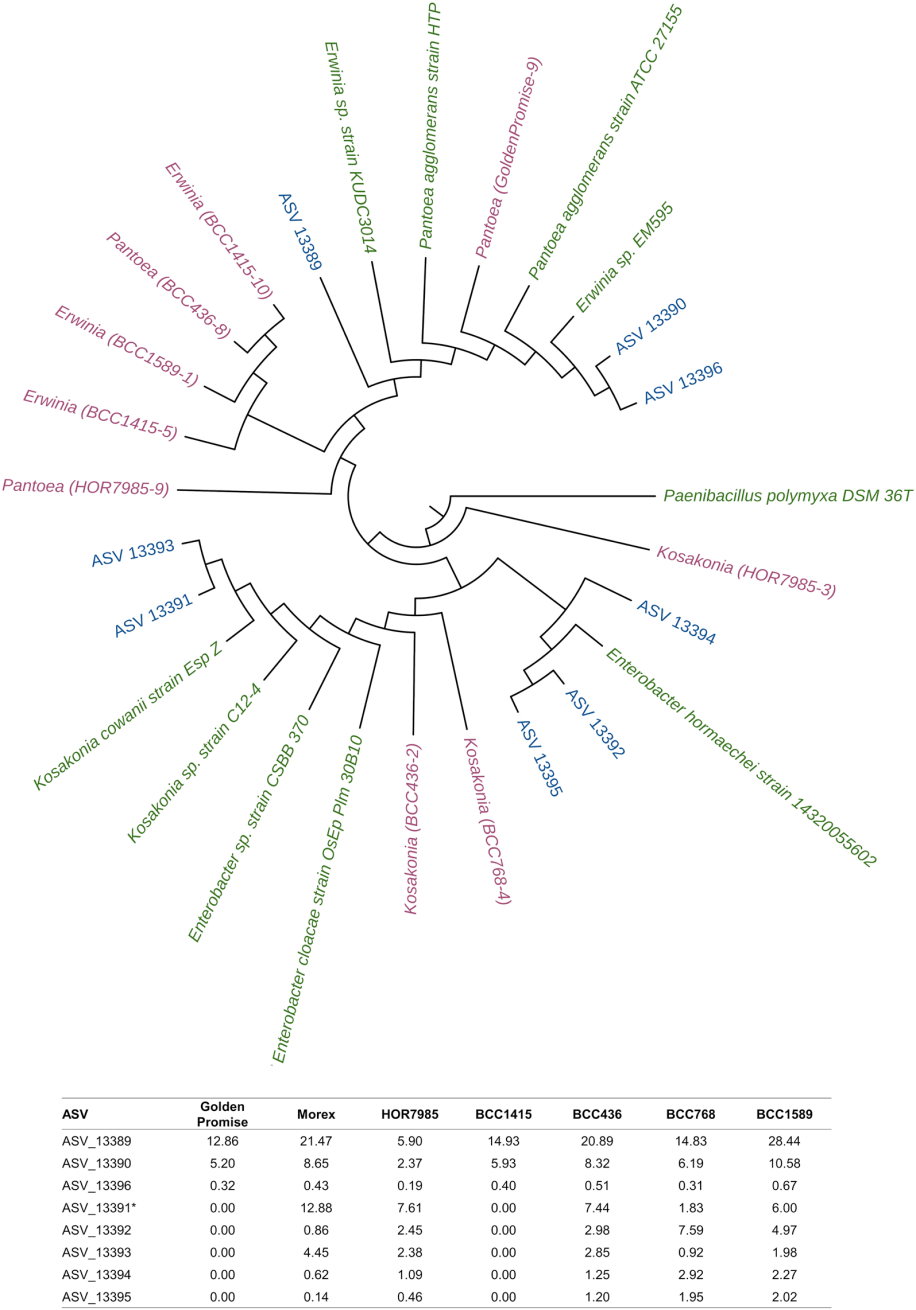
Phylogenetic analysis of *Enterobacteriaceae*-related 16S rRNA gene sequences of isolates, ASVs and NCBI reference strains. For the phylogenetic tree, all *Enterobacteriaceae*-related consensus sequences of isolated endophytes (nine; pink) and the most abundant unclassified *Enterobacteriaceae* ASVs (eight; blue) were used. Additionally, reference strains were chosen from NCBI database (green). The phylogenetic tree topology was obtained by UPGMA cluster and based on distance methods. *Paenibacillus polymyxa* DSM 36 T was used as the outgroup organism. Below the phylogenetic tree, relative abundances of *Enterobacteriaceae*-related ASVs are shown, the * highlights the ASV that was closely affiliated to the *Kosakonia* isolates obtained from HOR7985, BCC436 and BCC768 (see also Table 3)

For endophytic isolates belonging to the family *Enterobacteriaceae*, the comparison between ASV sequences and isolate consensus sequences resulted in mainly two ASV sequences of unclassified *Enterobacteriaceae*: one ASV sequence with high sequence identity to *Erwinia* and *Pantoea* isolates and one for *Kosakonia* according to NCBI. A phylogenetic tree (Fig. 5) including consensus sequences from endophytic isolates, ASVs of the eight most abundant unclassified *Enterobacteriaceae* and NCBI reference strains of *Pantoea*, *Erwinia*, *Kosakonia* and *Enterobacter* was performed due to the high relative abundance of unclassified *Enterobacteriaceae* in barley seeds (Table 2). The phylogenetic tree clarified the taxonomic affiliation of *Enterobacteriaceae*-related isolate and ASV sequences towards two main clusters: one cluster affiliated to *Pantoea* and *Erwinia* reference sequences, and one cluster affiliated to *Kosakonia* and *Enterobacter* reference sequences.

The reference genotype Golden Promise was chosen to identify endophytic isolates that can be easily enriched in non-selective media. After overnight enrichment of seed paste, only isolates affiliated to two different species, namely *Pantoea* and *Paenibacillus* were enriched.

### In vitro functional characterization of barley seed endophytes for potential plant beneficial activities

We hypothesized that barley endophytes produce secondary metabolites that may influence plant physiology, e.g. the capacity of enhancing plant defense. These plant beneficial activities can be of importance for improving alternative agricultural practices. Therefore we assessed lytic enzyme activity and secondary metabolites with potential plant defense enhancing capacity via different bioassays (Table 3). Our results revealed that all *Curtobacterium* isolates (15) displayed protease activity. Cellulase activity and IAA production were detected in most of these isolates (13 and 13, respectively). The β-1,3-glucanase activity varied in its appearance and intensity among the *Curtobacterium* isolates.

All 15 *Paenibacillus* isolates from different plant genotypes displayed protease, β-1,3-glucanase and cellulase activity. Chitinase activity varied between the different isolates. All *Paenibacillus* isolates were able to produce IAA.

Most of the *Sanguibacter* isolates (6) showed β-1,3-glucanase activity. One of the isolates was able to degrade cellulose and another one chitin. Four *Sanguibacter* isolates were shown to be able to use ACC as alternative nitrogen source.

All five *Saccharibacillus* isolates displayed cellulase activity, but only four isolates displayed β-1,3-glucanase activity and three ACC deaminase activity.

All isolates of *Pantoea* (3), *Erwinia* (3) and *Kosakonia* (3) produced siderophores and IAA and were able to solubilize phosphate. The only isolates that produced AHLs belonged to *Pantoea* (3) and *Erwinia* (2).

In summary, our results supported that barley seed endophytes possessed several characteristics with potential plant beneficial or plant resistance inducing potential.

### Rhizosphere microbiota of the barley 7’set in early growth stage

We aimed to investigate how the barley genotype and its seed microbiome influence the rhizosphere microbiome. The composition of the early rhizosphere microbiome of the barley 7’set was investigated in an agricultural soil with contrasting tillage practice. We chose a soil managed with the two different tillage practices mouldboard plough (MP) and cultivator tillage (CT) as model to analyze if the usage of different genotypes of barley would have any effect in different agricultural backgrounds. 16S rRNA gene amplicon sequencing obtained 1,487,732 reads assigned to 14,460 ASVs. The median read number was 21,737.5 reads per sample. The rarefaction curves (Additional file 1: Fig. S5) showed that the sequence library size was sufficient to cover the microbial diversity in each sample.

#### Barley seed microbiome shared only few genera with the rhizosphere microbiome

**Fig. 6:**
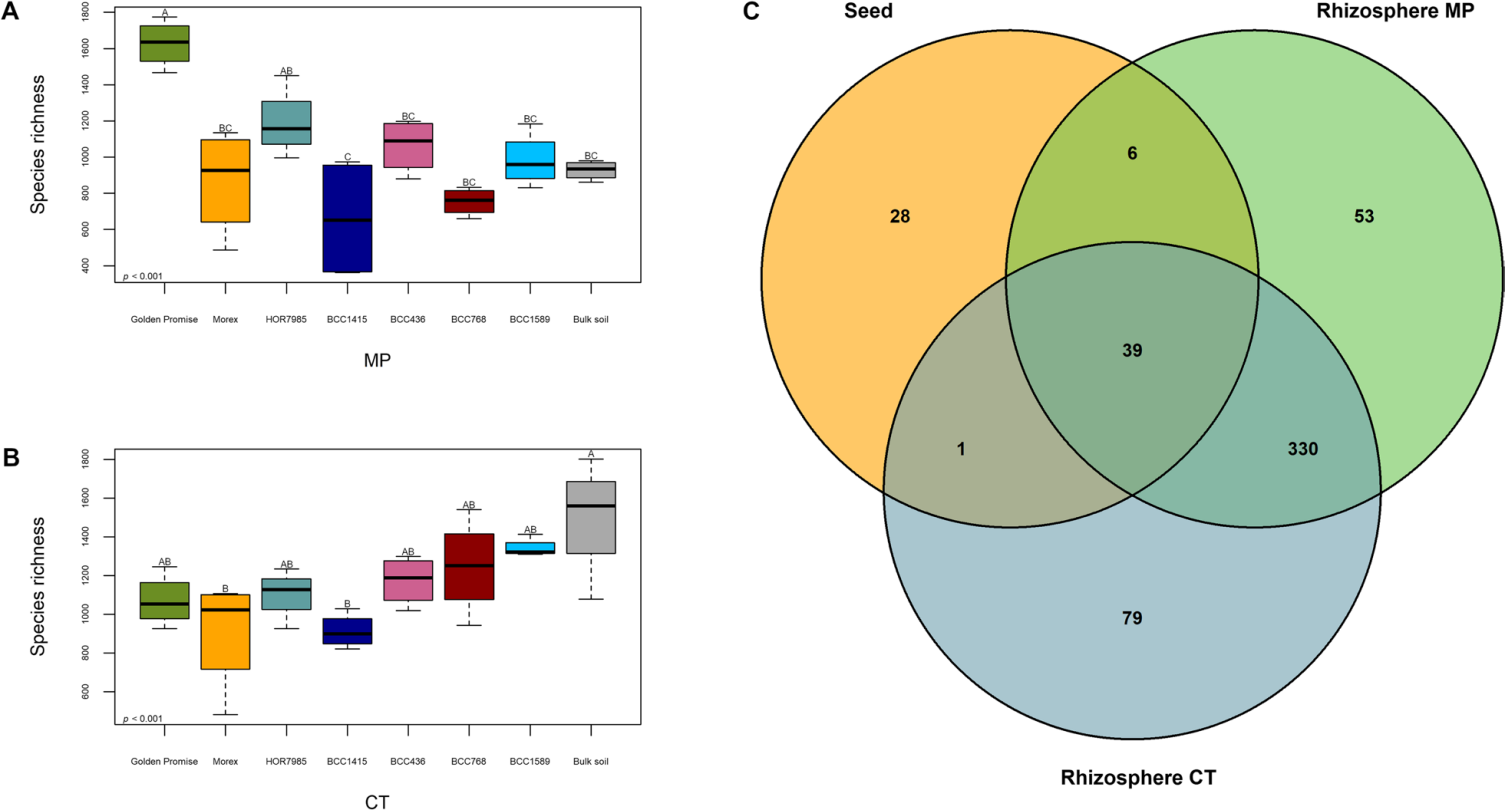
Rhizomicrobiota were influenced by the genotype and shared few genera with the seed microbiota. Species richness of bulk soil and barley rhizosphere microbiome of seven different genotypes were harvested from the two soil variants MP (mouldboard plough; **a**) and CT (cultivator tillage; **b**). The different genotypes revealed varying Species richness depending on the barley genotype. ANOVA confirmed a significant influence of the genotype (*p* ≤ 0.001). Values and statistics can be found in Additional file 1: Table S4 and S5. The Venn diagram revealed 39 shared genera between the barley seed and both MP and CT rhizosphere microbiota **c** of all barley genotypes

The endophytic seed microbiome shared 39 genera with both MP and CT rhizosphere (Fig. 6c). Additionally, six genera and one genus were shared with only MP or CT rhizosphere, respectively. The 39 shared genera belonged for instance to unclassified *Enterobacteriaceae, Burkholderiaceae*, *Microbacteriaceae*, *Paenibacillus, Sphingomonas*, *Massilia*, *Pseudomonas, Bacillus, Rhizobium* and *Stenotrophomonas*. Interestingly, the genera *Sphingomonas*, unclassified *Burkholderiaceae*, *Massilia* and *Bacillus* had similar relative abundances in both seed and rhizosphere microbiome. In contrast, members like the unclassified *Enterobacteriaceae* or *Stenotrophomonas* occurred in very low abundances in the rhizosphere.

#### Differences in rhizosphere microbiome diversity between the barley genotypes

Pronounced differences in microbial alpha-diversity of the rhizosphere microbiome among the plant genotypes were revealed for Species richness (*p* ≤ 0.001; Fig. 6a, b). The values ranged between 659.07 and 1625.07 (Additional file 1: Tables S4 and S5). The microbial beta-diversity of rhizosphere samples was visualized by NMDS separately for rhizosphere samples of MP and CT (Additional file 1: Fig. S7A, B). For both soil variants, the rhizosphere community composition was significantly influenced by the plant genotype (ANOSIM *p* ≤ 0.001), however, the different genotypes appeared in narrow clusters.

The influence of the plant genotype on the rhizosphere microbiome was further confirmed by PERMANOVA (*R*^2^ = 0.33, *p* ≤ 0.001 for MP and *R*^2^ = 0.26, *p* ≤ 0.001 for CT; Table 4). However, pairwise comparison revealed only significant differences between some genotypes (Additional file 1: Tables S6 and S7). The microhabitat rhizosphere versus bulk soil had only minor influence (MP: *R*^2^ = 0.06, *p* ≤ 0.01; CT: *R*^2^ = 0.06, *p* ≤ 0.001; Table 4).

**Table 4:** PERMANOVA of barley rhizosphere and bulk soil samples

PERMANOVA	*R*^2^	*p* ≤
Management MP versus CT	0.08	0.001
Bulk soil MP versus CT	0.27	0.05
MP BS versus RS	0.06	0.01
MP RS genotype	0.33	0.001
CT BS versus RS	0.06	0.001
CT RS genotype	0.26	0.001

Rhizosphere (RS) and bulk soil (BS) samples were obtained from seven different genotypes and an agricultural soil with two diverse tillage practices (mouldboard plough: MP versus cultivator tillage: CT)

#### Genotype-dependent rhizomicrobiota is also influenced by the tillage practice

Interestingly, Species richness of the different genotypes was also depending on the tillage practice (tillage practice *p* ≤ 0.01; tillage practice: genotype *p* ≤ 0.001). In MP soil, Golden Promise had the highest value for Species richness (1625.07; Additional file 1: Table S4), whereas in CT soil, Golden Promise had a lower value (1068.03) and the highest value was detected for BCC1589 (1339.95; Additional file 1: Table S5). The Shannon index showed a similar trend as observed for Species richness (Additional file 1: Fig. S6). Comparing the two contrasting tillage practices, Species richness and Shannon index showed higher values for CT bulk soil samples compared to MP. Microbial beta-diversity was influenced by the tillage practice as well (*R*^2^ = 0.08, *p* ≤ 0.001; Table 4).

Analysis of the taxonomic composition indicated that the most abundant phyla were Acidobacteria, Actinobacteria, Proteobacteria, Planctomycetes, Chloroflexi, Thaumarchaeota, Verrucomicrobia and Bacteroidetes for barley rhizosphere and bulk soil samples of the two soil variants MP and CT (Additional file 1: Table S8). Genotype-dependent differences on phylum level were observed to depend on the soil variant. Bacteroidetes had a higher relative abundance in the MP rhizosphere of Golden Promise (4.05%) compared to BCC1415 (2.06%). This trend was not visible for CT rhizosphere, but the proportion of Bacteroidetes in both bulk soils had a similar level. Actinobacteria reached similar relative abundances in both soil variants for Morex (20.03–20.25%) and HOR7985 (19.33–20.09%), but compared to those, they occurred in higher relative abundance in CT compared to MP rhizosphere for BCC768 and BCC1589 (23.68% versus 18.14% and 23.17% versus 17.27%, respectively). Highly abundant genera in rhizosphere and bulk soil of the two soil variants belonged to *Subgroup 6*, *Nitrososphaeraceae* and *Sphingomonas* (Additional file 1: Table S9).

Although the barley rhizosphere microbiome was highly influenced by the plant genotype as shown by alpha- and beta-diversity analyses, the taxonomic composition and highly abundant genera were also influenced by the management practice of the agricultural soil. Nevertheless, the seed and rhizosphere microbiota shared few genera.

## Discussion

Managing the plant microbiome to improve plant health could be a key for a more sustainable agriculture [64]. During the last years more and more research focused on the indigenous seed microbiome and its potential beneficial members driving plant’s tolerance to biotic and abiotic stress factors [65–67]. Seed endophytes that successfully colonize seedlings have been suggested to be important research targets for improvement of seed treatment technologies [68].

In the present study, we demonstrated that the genotype of barley had a significant influence on the seed and rhizosphere microbiome. However, few members of the seed microbiome were actually detected in the rhizosphere. An influence of the genotype on the barley seed microbiome was already reported in an RNA-based study by Yang et al. [21]. However, in their study, nothing was known about the genetic relatedness of the different barley cultivars used [21]. In our study, we used five genotypes of barley that were proposed to present the genetic diversity within a 224 spring barley accession set [14]. Although the different seed microbiomes were influenced by the genotype, no correlation was observed between the seed microbiome and the genetic variation in SNPs, suggesting that the seed microbiome is at least not determined by the SNPs investigated. Future studies for plant breeding research could explore quantitative trait loci (QTL) related to seed microbial diversity as already shown for maize leaf epiphytic bacteria [69].

The study of Wehner et al. [14] exploring the same barley 7’set additionally investigated the priming efficiency of the different genotypes when primed with *Ensifer meliloti* expR^+^ against the fungal leaf pathogen *Puccinia hordei*. HOR7985 was the best primable genotype [14] and interestingly, this genotype showed the highest seed microbiome diversity in the present study (Fig. 1a, b; Additional file 1: Table S2). High microbial diversity inside seeds may positively shape future interactions with plant beneficial microorganisms. In accordance with this assumption, genotype BCC436 that was not primable by *E. meliloti* expR^+^ [14] showed low microbial alpha-diversity (Fig. 1a, b; Additional file 1: Table S2).

The seed microbiome of genotype BCC1415 that was grown at the same field site in Germany as all other genotypes (except Golden Promise) was most different in beta-diversity, but surprisingly not the seed microbiome of Golden Promise grown in the UK (Fig. 1c; Table 1). The high impact of the genotype on the seed microbiome structure was proven by exclusion of Golden Promise from the analysis for both alpha- and beta-diversity (Additional file 1: Fig. S2). Although an influence of the soil on the establishment of the endophytic seed microbiome was reported before [70], it seems that the genotype represents higher impact on the seed microbiome. Previous studies reported similar results for the rice seed microbiome and the sugar beet seed microbiome showing that the genotype had a higher influence on the seed microbiome than the geographic location [71, 72].

Although different cultivars and genotypes were used, the three studies on the barley seed microbiome based on RNA [21] and DNA [5, present study], found that *Pseudomonas* and *Stenotrophomonas* were prevalent in the seed microbiome, however, in varying relative abundances. In both DNA-based studies, *Enterobacteriaceae* were dominant members in barley seeds [5, present study], while in the RNA-based study, *Phyllobacteriaceae* were highly abundant, possibly due to the fact that the seed microbiome was activated on R2A [21]. Despite the different techniques, *Paenibacillus* and *Saccharibacillus* were main genera in barley seeds [21, present study], whereas *Sanguibacter* and *Curtobacterium* were not detected by Yang et al. [21], suggesting that these genera might have been metabolically inactive or of lower relative abundance.

The dominant bacterial phyla, Proteobacteria, Actinobacteria and Firmicutes, observed in the barley endophytic seed microbiome in the present study, were previously reported from barley seed [5]. These phyla were also observed to be predominant in the rice seed microbiome [73, 74], in the wild cabbage seed microbiome [75], in pumpkin seed microbiome [76], as well as in seeds of *Brassicaceae* [77]. Interestingly, abundant genera in the present study such as *Pantoea*, *Paenibacillus*, *Kosakonia*, *Microbacterium*, *Pseudomonas*, *Curtobacterium* and *Erwinia* were also found in rice seeds [73, 74, 78], while *Sanguibacter*, *Saccharibacillus*, *Sphingomonas*, *Stenotrophomonas* and also *Microbacterium* and *Paenibacillus* were reported from tobacco seeds [79]. Since those phyla and even genera were found in seeds of numerous plant species, including dicots and monocots, we suggest that bacteria affiliated to these phyla belong to a universal core seed microbiome. This core microbiome would be adapted to the plant compartment and be present in seeds regardless of the plant species, origin, physiology or metabolism. The proportion of the members of the core seed microbiome seems to vary in relative abundance according to the plant species and genotype, soil type or geographic location.

Although the most dominant ASVs in the present study were affiliated to unclassified *Enterobacteriaceae*, the taxonomic affiliation of highly abundant *Enterobacteriaceae*-related ASVs was resolved by comparative 16S rRNA gene sequence analysis with reference strains and endophytic isolates (Fig. 5). A high proportion of the respective ASVs shared high sequence identity with different plant beneficial strains of *Pantoea agglomerans* [80]. Other ASVs were affiliated to *Enterobacter* and strains belonging to this genus were previously reported to harbor plant beneficial isolates [72, 81, 82]. *Enterobacteriaceae* were also found to be highly abundant in seeds of other plant species [76, 79] and we assume that they might contribute to their relative high abundance in the phyllosphere [83].

Excitingly, endophytes isolated from barley seeds in the present study shared high 16S rRNA gene sequence identity with dominant members of the core seed microbiome (Table 3) thus allowing insights into the potential functions of the respective members of the core seed microbiome. The diversity of isolates obtained from seeds of each plant genotype did not mirror the Species richness determined for the respective seed microbiome. Unexpectedly, *Pseudomonas* and *Rhizobium* being abundant members in the seed microbiome were missing in our isolate collection. This might be due to the cultivation method used while also a reduced cultivability due to environmental stress may play a role.

The plant beneficial traits tested in the present study (cell wall-degrading enzymes, ACC deaminase, phytohormone synthesis, siderophores and AHL production) were proposed to be common functions in the plant endophytic community [82, 84]. Isolates affiliated to *Paenibacillus* might be good candidates for biocontrol of phytopathogens or as plant growth promoting bacteria [85], as these isolates showed many beneficial activities (Table 3). Only isolates affiliated to *Pantoea*, *Erwinia* and *Kosakonia* showed siderophore production and phosphate solubilization. Isolates affiliated to these genera are well-known plant beneficial bacteria. In a previous study, the relative abundance of the plant pathogen *Xanthomonas campestris* pv. *vitians* was found to be negatively correlated with the appearance of *Pantoea* and *Erwinia* in lettuce phyllosphere, suggesting an important role for plant protection [86]. In the current study, most of the *Curtobacterium* isolates were affiliated to the species *C. flaccumfaciens* and only one isolate was affiliated to the species *C. herbarum.* The latter strain displayed a functional profile distinct from the other *Curtobacterium* isolates (Table 3). Previously, the genus *Curtobacterium* was reported as endophyte in various plant species [87–89]. Different *C. flaccumfaciens* strains were reported for their biocontrol activity [90–93]. Additionally, *C. herbarum* CS10 showed siderophore and IAA production and plant growth promoting activity [94]. The knowledge of plant beneficial traits of *Sanguibacter* species is scarce, but this genus was reported as endophyte before [89, 93]. *Sanguibacter* isolates from barley seeds in the present study were capable of ACC deaminase and ß-1,3-glucanase. Isolates affiliated to *Saccharibacillus* were previously obtained from barley seed [5] and other plant species [95–97], but only in the present study the potentially plant beneficial functions of *Saccharibacillus* isolates (β-1,3-glucanase, cellulase, ACC deaminase, IAA) were determined. Seed endophytes belonging to these genera offer the potential to study their plant beneficial traits and the ability to induce resistance.

The indigenous seed microbiome harbors endophytes that are proposed as synergetic components of the innate plant immunity, as the rice seed endophyte *Sphingomonas* was able to confer resistance against *Burkholderia plantarii* in later plant development [74]. Ubiquitous taxa in wheat seedlings were found to derive from the seed microbiome, but also the soil microbiome had an important but variable influence [68]. A previous study on the barley seed microbiome observed that the contribution of the seed microbiome to the endophytic root microbiome changed with increasing plant development and seed endophytes became less dominant in the endophytic root microbiome [21]. In the present study, we observed that the soil had a high influence on the barley rhizosphere microbiome composition, as the observed taxonomic composition and the relative abundances of genera did not show pronounced differences between the seven genotypes and were similar to soil and rhizosphere samples of previous studies focusing on the same field soils [29, 98]. Most of the highly abundant seed endophytes were low in relative abundance (*Stenotrophomonas*) or not detected (*Curtobacterium, Pantoea*, *Sanguibacter*) in barley rhizosphere samples. Nevertheless, some genera such as *Paenibacillus*, *Pseudomonas* or *Sphingomonas* were shared between seed and rhizosphere microbiome, but their relative abundances differed between the microhabitats.

Interestingly, the influence of the plant genotype on the rhizosphere microbiome was dependent on the previous soil management, as displayed by the alpha-diversity indices (Additional file 1: Tables S4, S5 and Fig S4). The soil with two different tillage histories (mouldboard plough versus cultivator tillage) had different physicochemical characteristics [29] suggesting that the interplay between soil nutrients and the plant might be important for the rhizosphere microbiome assembly.

## Conclusion

Although a plant genotype-dependent endophytic seed microbiome was found in the present study, no clear correlation was observed between the seed microbiome and the genetic variation among the different genotypes. Nevertheless, a core microbiome common for all genotypes was observed. Endophytic bacteria that were shown to induce plant resistance in previous studies, belonged to genera that were highly abundant in the seeds. Excitingly, isolates affiliated to these genera were obtained from barley seeds in the present study. Most of the isolated endophytes showed diverse plant beneficial characteristics in vitro. Our endophytic isolates belonged to genera such as *Paenibacillus*, *Pantoea* and *Curtobacterium* that contain isolates influencing plant physiology, but also to genera such as *Sanguibacter* or *Saccharibacillus* not known for plant beneficial traits. Whether these strains have a plant beneficial influence in vivo still needs to be elucidated. Although a plant genotype-dependent rhizosphere microbiome composition was detected, the contribution of the seed microbiome was only minor, but some members of the seed microbiome were also detected in the rhizosphere microbiome. As seed endophytes may play an important role in defense priming, we propose that future breeding strategies should consider genotypes with high abundance and diversity of plant beneficial microbes.

## Supplementary Information

**Additional file 1**. **Table S1**. Primer sequences. **Table S2**. Pielou’s evenness, Species richness and Shannon index of endophytic seed microbial communities obtained from seven different barley genotypes. **Table S3**. Dominant phyla in the endophytic seed microbiome of seven different barley genotypes. **Table S4**. Pielou’s evenness, Species richness and Shannon index of bulk soil and barley rhizosphere samples from seven different genotypes grown in mouldboard plough (MP) soil. **Table S5**. Pielou’s evenness, Species richness and Shannon index of bulk soil and barley rhizosphere samples from seven different genotypes grown in cultivator tillage (CT) soil. **Table S6**. PERMANOVA of each barley genotype of rhizosphere samples obtained from plants grown in mouldboard plough (MP) soil. **Table S7**. PERMANOVA of each versus each barley genotype of rhizosphere samples obtained from plants grown in cultivator tillage (CT) soil. **Table S8**. Dominant phyla in bulk soil and barley rhizosphere of seven different genotypes grown in two diverse soils (MP; CT). **Table S9**. Twenty most abundant genera in bulk soil and barley rhizosphere of seven different genotypes grown in two diverse soils (MP; CT). **Figure S1**. Rarefaction curves of 16S rRNA gene amplicon sequencing data of the barley seed microbiome. **Figure S2**. Microbial diversity of the endophytic seed microbiome varied between six barley genotypes originated from the same place. The microbial alpha-diversity indices Species richness (A) and Shannon diversity index (B) varied depending on the plant genotype (ANOVA for Species richness *p* ≤ 0.001 and Shannon index *p* ≤ 0.01). Beta-diversity of the endophytic seed microbiome was visualized by NMDS (C). The microbiome composition based on Bray-Curtis community dissimilarities (ASVs obtained from 16S rRNA gene amplicon sequencing) and was assessed from DNA of surface-sterilized barley seeds of six genotypes harvested at the same field site. **Figure S3**. Logarithm of colony forming units (CFUs)/g seed of seven different barley genotypes determined after 48 h incubation at 28 °C. 0.5 g of surface-sterilized seeds were ground and solved in 4.5 mL sterile double-distilled water. The seed suspension was plated on R2A supplemented with 100 µg/mL cycloheximide. **Figure S4**. BOX fingerprint of isolated endophytes taxonomically affiliated to the genus *Curtobacterium*. **Figure S5**. BOX fingerprint of isolated endophytes taxonomically affiliated to the genus *Paenibacillus*. **Figure S6**. Rarefaction curves of 16S rRNA gene amplicon sequencing data of barley rhizosphere and bulk soil samples from two different soils (MP: mouldboard plough; CT: cultivator tillage) and seven different genotypes. **Figure S7**. Shannon index of bulk soil and barley rhizosphere samples of seven different genotypes grown in MP and CT soils. **Figure S8**. Non-metric multidimensional scaling (NMDS) of barley rhizosphere microbial communities from seven different genotypes grown in MP (A) and CT (B) soil. The rhizosphere community composition based on Bray-Curtis dissimilarities and was obtained from ASVs. The seven different genotypes were grown in MP (mouldboard plough) and CT (cultivator tillage) soil until BBCH13. Respective bulk soil samples are not shown. ANOSIM verified significant differences between the genotypes (*p* ≤ 0.001)

## Data Availability

Raw data of 16S rRNA gene amplicon sequences supporting the findings of the present study are available in the Sequence Read Archive of NCBI under BioProject accession PRJNA727806.

## Abbreviations

ACC: 1-Aminocyclopropanecarboxylic acid
AHLs: *N*-acyl homoserin lactones
ANOSIM: Analysis of similarities
ANOVA: Analysis of variance
ASVs: Amplicon sequence variants
BPW: Buffered peptone water
CFUs: Colony forming units
CT: Cultivator tillage
2,4-DAPG: 2,4-Diacetylphloroglucinol
DMSO: Dimethyl sulfoxide
dNTP: Deoxyribonucleotide triphosphate
IAA: Indole-3-acetic acid
ISR: Induced systemic resistance
MP: Mouldboard plough
NMDS: Non-metric multidimensional scaling
OTUs: Operational taxonomic units
PERMANOVA: Permutational analysis of variance
QTL: Quantitative trait loci
SNPs: Single nucleotide polymorphisms

## References

[1] Savary S, Willocquet L, Pethybridge SJ, Esker P, McRoberts N, Nelson A (2019). The global burden of pathogens and pests on major food crops. Nat Ecol Evol.

[2] Hillocks RJ (2012). Farming with fewer pesticides: EU pesticide review and resulting challenges for UK agriculture. Crop Prot.

[3] Berg G, Raaijmakers JM (2018). Saving seed microbiomes. ISME J.

[4] Shahzad R, Khan AL, Bilal S, Asaf S, Lee I-J (2018). What is there in seeds? vertically transmitted endophytic resources for sustainable improvement in plant growth. Front Plant Sci.

[5] Rahman MM, Flory E, Koyro H-W, Abideen Z, Schikora A, Suarez C (2018). Consistent associations with beneficial bacteria in the seed endosphere of barley (*Hordeum vulgare* L.). Syst Appl Microbiol.

[6] Newton AC, Flavell AJ, George TS, Leat P, Mullholland B, Ramsay L (2011). Crops that feed the world 4. Barley: a resilient crop? Strengths and weaknesses in the context of food security. Food Sec..

[7] Mascher M, Gundlach H, Himmelbach A, Beier S, Twardziok SO, Wicker T (2017). A chromosome conformation capture ordered sequence of the barley genome. Nature.

[8] Schreiber M, Mascher M, Wright J, Padmarasu S, Himmelbach A, Heavens D (2020). A genome assembly of the barley ‘Transformation Reference’ Cultivar Golden Promise. G3 Bethesda..

[9] Jayakodi M, Padmarasu S, Haberer G, Bonthala VS, Gundlach H, Monat C (2020). The barley pan-genome reveals the hidden legacy of mutation breeding. Nature.

[10] Monat C, Schreiber M, Stein N, Mascher M (2019). Prospects of pan-genomics in barley. Theor Appl Genet.

[11] Oerke E-C, Dehne H-W, Schönbeck F, Weber A (1994). Crop production and crop protection: estimated losses in major food and cash crops.

[12] Oerke E-C (2006). Crop losses to pests. J Agric Sci.

[13] Ramírez-Carrasco G, Martínez-Aguilar K, Alvarez-Venegas R (2017). Transgenerational defense priming for crop protection against plant pathogens: a hypothesis. Front Plant Sci.

[14] Wehner G, Kopahnke D, Richter K, Kecke S, Schikora A, Ordon F (2019). Priming is a suitable strategy to enhance resistance towards leaf rust in barley. Phytobiomes J.

[15] Walters DR, Ratsep J, Havis ND (2013). Controlling crop diseases using induced resistance: challenges for the future. J Exp Bot.

[16] Pieterse CMJ, Zamioudis C, Berendsen RL, Weller DM, van Wees SCM, Bakker PAHM (2014). Induced systemic resistance by beneficial microbes. Annu Rev Phytopathol.

[17] Mauch-Mani B, Baccelli I, Luna E, Flors V (2017). Defense priming: an adaptive part of induced resistance. Annu Rev Plant Biol.

[18] Zamioudis C, Pieterse CMJ (2012). Modulation of host immunity by beneficial microbes. Mol Plant Microbe Interact.

[19] Vannier N, Agler M, Hacquard S (2019). Microbiota-mediated disease resistance in plants. PLOS Pathog.

[20] Han S, Li D, Trost E, Mayer KF, Vlot AC, Heller W (2016). Systemic responses of barley to the 3-hydroxy-decanoyl-homoserine lactone producing plant beneficial endophyte *Acidovorax radicis* N35. Front Plant Sci.

[21] Yang L, Danzberger J, Schöler A, Schröder P, Schloter M, Radl V (2017). Dominant groups of potentially active bacteria shared by barley seeds become less abundant in root associated microbiome. Front Plant Sci.

[22] Walters DR, Havis ND, Paterson L, Taylor J, Walsh DJ (2011). Cultivar effects on the expression of induced resistance in spring barley. Plant Dis.

[23] Shrestha A, Elhady A, Adss S, Wehner G, Böttcher C, Heuer H (2019). Genetic differences in barley govern the responsiveness to *N* -acyl homoserine lactone. Phytobiomes J.

[24] Pasam RK, Sharma R, Malosetti M, van Eeuwijk FA, Haseneyer G, Kilian B, Graner A (2012). Genome-wide association studies for agronomical traits in a world wide spring barley collection. BMC Plant Biol.

[25] Kutter S, Hartmann A, Schmid M (2006). Colonization of barley (*Hordeum vulgare*) with *Salmonella enterica* and *Listeria* spp. FEMS Microbiol Ecol.

[26] Datukishvili N, Gabriadze I, Kutateladze T, Karseladze M, Vishnepolsky B (2010). Comparative evaluation of DNA extraction methods for food crops. Int J Food Sci Technol.

[27] Deubel A, Hofmann B, Orzessek D (2011). Long-term effects of tillage on stratification and plant availability of phosphate and potassium in a loess chernozem. Soil Tillage Res.

[28] Meier U. Growth stages of mono- and dicotyledonous plants: BBCH Monograph: open Agrar Repositorium; 2018.

[29] Bziuk N, Maccario L, Douchkov D, Lueck S, Babin D, Sørensen SJ, et al. Tillage shapes the soil and rhizosphere microbiome of barley - but not its susceptibility towards *Blumeria graminis* f. sp. *hordei*. FEMS Microbiol Ecol. 2021. 10.1093/femsec/fiab018. 10.1093/femsec/fiab018 33544837

[30] Moronta-Barrios F, Gionechetti F, Pallavicini A, Marys E, Venturi V (2018). Bacterial microbiota of rice roots: 16S-based taxonomic profiling of endophytic and rhizospheric diversity, endophytes isolation and simplified endophytic community. Microorganisms.

[31] Vestheim H, Jarman SN (2008). Blocking primers to enhance PCR amplification of rare sequences in mixed samples: a case study on prey DNA in Antarctic krill stomachs. Front Zool.

[32] Arenz BE, Schlatter DC, Bradeen JM, Kinkel LL (2015). Blocking primers reduce co-amplification of plant DNA when studying bacterial endophyte communities. J Microbiol Methods.

[33] Martin M (2011). Cutadapt removes adapter sequences from high-throughput sequencing reads. EMBnet J.

[34] Callahan BJ, McMurdie PJ, Rosen MJ, Han AW, Johnson AJA, Holmes SP (2016). DADA2: High-resolution sample inference from Illumina amplicon data. Nat Methods.

[35] Bolyen E, Rideout JR, Dillon MR, Bokulich NA, Abnet CC, Al-Ghalith GA (2019). Reproducible, interactive, scalable and extensible microbiome data science using QIIME 2. Nat Biotechnol.

[36] Quast C, Pruesse E, Yilmaz P, Gerken J, Schweer T, Yarza P (2013). The SILVA ribosomal RNA gene database project: improved data processing and web-based tools. Nucleic Acids Res.

[37] McMurdie PJ, Holmes S (2013). phyloseq: an R package for reproducible interactive analysis and graphics of microbiome census data. PLOS ONE.

[38] Davis NM, Proctor DM, Holmes SP, Relman DA, Callahan BJ (2018). Simple statistical identification and removal of contaminant sequences in marker-gene and metagenomics data. Microbiome.

[39] Hothorn T, Bretz F, Westfall P (2008). Simultaneous inference in general parametric models. Biom J.

[40] Oksanen J, Blanchet FG, Friendly M, Kindt R, Legendre P, McGlinn D, et al. Vegan: community ecology package. 2019. https://CRAN.R-project.org/package=vegan. Accessed 15 Sep 2020.

[41] Chen H. VennDiagram: generate high-resolution Venn and Euler Plots. 2018. https://CRAN.R-project.org/package=VennDiagram. Accessed 3 Mar 2021.

[42] Dusa A. Venn: draw Venn diagrams. 2020. https://CRAN.R-project.org/package=venn. Accessed 3 Mar 2021.

[43] Comadran J, Kilian B, Russell J, Ramsay L, Stein N, Ganal M (2012). Natural variation in a homolog of antirrhinum CENTRORADIALIS contributed to spring growth habit and environmental adaptation in cultivated barley. Nat Genet.

[44] Milner SG, Jost M, Taketa S, Mazón ER, Himmelbach A, Oppermann M (2019). Genebank genomics highlights the diversity of a global barley collection. Nat Genet..

[45] Jombart T (2008). adegenet: a R package for the multivariate analysis of genetic markers. Bioinformatics.

[46] Kamvar ZN, Tabima JF, Grünwald NJ (2014). Poppr: an R package for genetic analysis of populations with clonal, partially clonal, and/or sexual reproduction. PeerJ.

[47] Hijmans RJ. Geosphere: spherical trigonometry. 2019. https://CRAN.R-project.org/package=geosphere. Accessed 26 Mar 2021.

[48] Martin B, Humbert O, Camara M, Guenzi E, Walker J, Mitchell T (1992). A highly conserved repeated DNA element located in the chromosome of *Streptococcus pneumoniae*. Nucleic Acids Res.

[49] Heuer H, Kopmann C, Binh CTT, Top EM, Smalla K (2009). Spreading antibiotic resistance through spread manure: characteristics of a novel plasmid type with low %G+C content. Environ Microbiol.

[50] Katoh K, Standley DM (2013). MAFFT multiple sequence alignment software version 7: improvements in performance and usability. Mol Biol Evol.

[51] Afgan E, Baker D, Batut B, van den Beek M, Bouvier D, Cech M (2018). The Galaxy platform for accessible, reproducible and collaborative biomedical analyses: 2018 update. Nucleic Acids Res.

[52] Charif D, Lobry J, editors. SeqinR 1.0–2: a contributed package to the R project for statistical computing devoted to biological sequences retrieval and analysis. Berlin: Springer; 2007.

[53] Schliep K, Potts AJ, Morrison DA, Grimm GW (2017). Intertwining phylogenetic trees and networks. Methods Ecol Evol.

[54] Paradis E, Schliep K (2019). ape 5.0: an environment for modern phylogenetics and evolutionary analyses in R. Bioinformatics.

[55] Letunic I, Bork P (2007). Interactive tree of life (iTOL): an online tool for phylogenetic tree display and annotation. Bioinformatics.

[56] Camacho C, Coulouris G, Avagyan V, Ma N, Papadopoulos J, Bealer K, Madden TL (2009). BLAST+: architecture and applications. BMC Bioinform.

[57] Cock PJA, Chilton JM, Grüning B, Johnson JE, Soranzo N (2015). NCBI BLAST+ integrated into Galaxy. Gigascience.

[58] Weinert N, Meincke R, Gottwald C, Heuer H, Schloter M, Berg G, Smalla K (2010). Bacterial diversity on the surface of potato tubers in soil and the influence of the plant genotype. FEMS Microbiol Ecol.

[59] Berg G, Fritze A, Roskot N, Smalla K (2001). Evaluation of potential biocontrol rhizobacteria from different host plants of *Verticillium dahliae* Kleb. J Appl Microbiol.

[60] Nautiyal CS (1999). An efficient microbiological growth medium for screening phosphate solubilizing microorganisms. FEMS Microbiol Lett.

[61] Koo S-Y, Hong SH, Ryu HW, Cho K (2010). Plant growth-promoting trait of rhizobacteria isolated from soil contaminated with petroleum and heavy metals. J Microbiol Biotechnol.

[62] Schwyn B, Neilands JB (1987). Universal chemical assay for the detection and determination of siderophores. Anal Biochem.

[63] Durán N, Justo GZ, Durán M, Brocchi M, Cordi L, Tasic L (2016). Advances in *Chromobacterium violaceum* and properties of violacein: its main secondary metabolite: a review. Biotechnol Adv.

[64] Berg G, Köberl M, Rybakova D, Müller H, Grosch R, Smalla K (2017). Plant microbial diversity is suggested as the key to future biocontrol and health trends. FEMS Microbiol Ecol.

[65] Rybakova D, Mancinelli R, Wikström M, Birch-Jensen A-S, Postma J, Ehlers R-U (2017). The structure of the *Brassica napus* seed microbiome is cultivar-dependent and affects the interactions of symbionts and pathogens. Microbiome.

[66] Sánchez-López AS, Pintelon I, Stevens V, Imperato V, Timmermans J-P, González-Chávez C (2018). Seed endophyte microbiome of *Crotalaria pumila* unpeeled: identification of plant-beneficial Methylobacteria. Int J Mol Sci.

[67] Girsowicz R, Moroenyane I, Steinberger Y (2019). Bacterial seed endophyte community of annual plants modulated by plant photosynthetic pathways. Microbiol Res.

[68] Walsh CM, Becker-Uncapher I, Carlson M, Fierer N (2021). Variable influences of soil and seed-associated bacterial communities on the assembly of seedling microbiomes. ISME J.

[69] Balint-Kurti P, Simmons SJ, Blum JE, Ballaré CL, Stapleton AE (2010). Maize leaf epiphytic bacteria diversity patterns are genetically correlated with resistance to fungal pathogen infection. Mol Plant Microbe Interact.

[70] Rodríguez CE, Antonielli L, Mitter B, Trognitz F, Sessitsch A (2020). Heritability and functional importance of the *Setaria viridis* bacterial seed microbiome. Phytobiomes J.

[71] Wolfgang A, Zachow C, Müller H, Grand A, Temme N, Tilcher R, Berg G (2020). Understanding the impact of cultivar, seed origin, and substrate on bacterial diversity of the sugar beet rhizosphere and suppression of soil-borne pathogens. Front Plant Sci.

[72] Walitang DI, Kim C-G, Jeon S, Kang Y, Sa T (2019). Conservation and transmission of seed bacterial endophytes across generations following crossbreeding and repeated inbreeding of rice at different geographic locations. Microbiologyopen.

[73] Raj G, Shadab M, Deka S, Das M, Baruah J, Bharali R, Talukdar NC (2019). Seed interior microbiome of rice genotypes indigenous to three agroecosystems of Indo-Burma biodiversity hotspot. BMC Genomics.

[74] Matsumoto H, Fan X, Wang Y, Kusstatscher P, Duan J, Wu S (2021). Bacterial seed endophyte shapes disease resistance in rice. Nat Plants.

[75] Tyc O, Putra R, Gols R, Harvey JA, Garbeva P (2020). The ecological role of bacterial seed endophytes associated with wild cabbage in the United Kingdom. Microbiologyopen.

[76] Adam E, Bernhart M, Müller H, Winkler J, Berg G (2018). The *Cucurbita pepo* seed microbiome: genotype-specific composition and implications for breeding. Plant Soil.

[77] Barret M, Bonneau S, Préveaux A (2015). Emergence shapes the structure of the seed microbiota. Appl Environ Microbiol.

[78] Walitang DI, Kim K, Madhaiyan M, Kim YK, Kang Y, Sa T (2017). Characterizing endophytic competence and plant growth promotion of bacterial endophytes inhabiting the seed endosphere of Rice. BMC Microbiol.

[79] Chen X, Krug L, Yang H, Li H, Yang M, Berg G, Cernava T (2020). *Nicotiana tabacum* seed endophytic communities share a common core structure and genotype-specific signatures in diverging cultivars. Comput Struct Biotechnol J.

[80] Walterson AM, Stavrinides J (2015). *Pantoea*: insights into a highly versatile and diverse genus within the *Enterobacteriaceae*. FEMS Microbiol Rev.

[81] Li H, Ding X, Wang C, Ke H, Wu Z, Wang Y (2016). Control of Tomato yellow leaf curl virus disease by *Enterobacter asburiae* BQ9 as a result of priming plant resistance in tomatoes. Turk J Biol.

[82] Sessitsch A, Hardoim P, Döring J, Weilharter A, Krause A, Woyke T (2012). Functional characteristics of an endophyte community colonizing rice roots as revealed by metagenomic analysis. Mol Plant Microbe Interact.

[83] Leff JW, Fierer N (2013). Bacterial communities associated with the surfaces of fresh fruits and vegetables. PLOS ONE.

[84] Liu H, Carvalhais LC, Crawford M, Singh E, Dennis PG, Pieterse CMJ, Schenk PM (2017). Inner plant values: diversity, colonization and benefits from endophytic bacteria. Front Microbiol.

[85] Rybakova D, Cernava T, Köberl M, Liebminger S, Etemadi M, Berg G (2016). Endophytes-assisted biocontrol: novel insights in ecology and the mode of action of *Paenibacillus*. Plant Soil.

[86] Rastogi G, Sbodio A, Tech JJ, Suslow TV, Coaker GL, Leveau JHJ (2012). Leaf microbiota in an agroecosystem: spatiotemporal variation in bacterial community composition on field-grown lettuce. ISME J.

[87] Bulgari D, Casati P, Brusetti L, Quaglino F, Brasca M, Daffonchio D, Bianco PA (2009). Endophytic bacterial diversity in grapevine (*Vitis vinifera* L.) leaves described by 16S rRNA gene sequence analysis and length heterogeneity-PCR. J Microbiol.

[88] Vega FE, Pava-Ripoll M, Posada F, Buyer JS (2005). Endophytic bacteria in *Coffea arabica* L. J Basic Microbiol.

[89] Herranen M, Kariluoto S, Edelmann M, Piironen V, Ahvenniemi K, Iivonen V (2010). Isolation and characterization of folate-producing bacteria from oat bran and rye flakes. Int J Food Microbiol.

[90] Raupach GS, Kloepper JW (1998). Mixtures of plant growth-promoting rhizobacteria enhance biological control of multiple cucumber pathogens. J Phytopathol.

[91] Raupach GS, Kloepper JW (2000). Biocontrol of cucumber diseases in the field by plant growth-promoting rhizobacteria with and without methyl bromide fumigation. Plant Dis.

[92] Lacava PT, Li W, Araújo WL, Azevedo JL, Hartung JS (2007). The endophyte *Curtobacterium flaccumfaciens* reduces symptoms caused by *Xylella fastidiosa* in *Catharanthus roseus*. J Microbiol.

[93] Jiang Z-K, Tuo Li, Huang D-L, Osterman IA, Tyurin AP, Liu S-W (2018). Diversity, novelty, and antimicrobial activity of endophytic Actinobacteria from mangrove plants in Beilun Estuary National Nature Reserve of Guangxi, China. Front Microbiol.

[94] Díez-Méndez A, Rivas R (2017). Improvement of saffron production using *Curtobacterium herbarum* as a bioinoculant under greenhouse conditions. AIMS Microbiol.

[95] Rivas R, García-Fraile P, Zurdo-Piñeiro JL, Mateos PF, Martínez-Molina E, Bedmar EJ (2008). *Saccharibacillus sacchari* gen. nov., sp. Nov., isolated from sugar cane. Int J Syst Evol Microbiol.

[96] Kämpfer P, Busse H-J, Kleinhagauer T, McInroy JA, Glaeser SP (2016). *Saccharibacillus endophyticus* sp. nov., an endophyte of cotton. Int J Syst Evol Microbiol.

[97] Besaury L, Remond C. Draft genome sequence of *Saccharibacillus* sp. Strain WB 17, isolated from wheat phyllosphere. Microbiol Resour Announc 2020. 10.1128/MRA.01201-19. 10.1128/MRA.01201-19 PMC7019059 32054704

[98] Babin D, Deubel A, Jacquiod S, Sørensen SJ, Geistlinger J, Grosch R, Smalla K (2019). Impact of long-term agricultural management practices on soil prokaryotic communities. Soil Biol Biochem.

