# Supplementary material for: The treasure inside barley seeds: microbial diversity and plant beneficial bacteria
Source: Environ Microbiome. 2021 Oct 28;16:20. doi: 10.1186/s40793-021-00389-8 (PMC8554914; doi:10.1186/s40793-021-00389-8)
Supplement: Supplementary file 1 — Additional file 1. Table S1. Primer sequences. Table S2. Pielou’s evenness, Species richness and Shannon index of endophytic seed microbial communities obtained from seven different barley genotypes. Table S3. Dominant phyla in the endophytic seed microbiome of seven different barley genotypes. Table S4. Pielou’s evenness, Species richness and Shannon index of bulk soil and barley rhizosphere samples from seven different genotypes grown in mouldboard plough (MP) soil. Table S5. Pielou’s evenness, Species richness and Shannon index of bulk soil and barley rhizosphere samples from seven different genotypes grown in cultivator tillage (CT) soil. Table S6. PERMANOVA of each barley genotype of rhizosphere samples obtained from plants grown in mouldboard plough (MP) soil. Table S7. PERMANOVA of each versus each barley genotype of rhizosphere samples obtained from plants grown in cultivator tillage (CT) soil. Table S8. Dominant phyla in bulk soil and barley rhizosphere of seven different genotypes grown in two diverse soils (MP; CT). Table S9. Twenty most abundant genera in bulk soil and barley rhizosphere of seven different genotypes grown in two diverse soils (MP; CT). Figure S1. Rarefaction curves of 16S rRNA gene amplicon sequencing data of the barley seed microbiome. Figure S2. Microbial diversity of the endophytic seed microbiome varied between six barley genotypes originated from the same place. The microbial alpha-diversity indices Species richness (A) and Shannon diversity index (B) varied depending on the plant genotype (ANOVA for Species richness p ≤ 0.001 and Shannon index p ≤ 0.01). Beta-diversity of the endophytic seed microbiome was visualized by NMDS (C). The microbiome composition based on Bray-Curtis community dissimilarities (ASVs obtained from 16S rRNA gene amplicon sequencing) and was assessed from DNA of surface-sterilized barley seeds of six genotypes harvested at the same field site. Figure S3. Logarithm of colony forming units (CFUs)/g seed of seve [file 40793_2021_389_MOESM1_ESM.pdf]

Table S1. Primer sequences.

Primer name	Primer sequence
16S-V4-515F	TCGTCGGCAGCGTCAGATGTGTATAAGAGACAGGTGCCAGCMGCCGCGGTAA
16S-V4-802R	GTCTCGTGGGCTCGGAGATGTGTATAAGAGACAGTACNVGGGTATCTAATCC
16S-V4-806R	GTCTCGTGGGCTCGGAGATGTGTATAAGAGACAGGACTACHVGGGTWTCTAAT
MitoBlk-515F	TCCCCATGCTTTCGCACCCCA/3SpC3/
CloroBlk-806R	GTCTCTAATCCCATTTGCTCC/3SpC3/

Table S2. Pielou’s evenness, Species richness and Shannon index of endophytic seed microbial communities obtained from seven different barley genotypes.

	**Golden Promise**	**Morex**	**HOR7985**	**BCC1415**	**BCC436**	**BCC768**	**BCC1589**
**Pielou's evenness**	0.82±0.07a	0.71±0.10a	0.86±0.04a	0.84±0.06a	0.73±0.05a	0.76±0.09a	0.69±0.08a
**Species richness**	75.69±21.47a	41.24±13.24b	81.19±18.18a	31.19±8.15b	41.12±4.49b	52.03±13.56ab	25.73±7.24b
**Shannon index**	3.53±0.56ab	2.62±0.58bc	3.75±0.35a	2.87±0.34abc	2.71±0.21bc	2.99±0.47abc	2.23±0.38c

Letters indicate significant differences (Tukey's test $p \leq 0.05$).

Table S3. Dominant phyla in the endophytic seed microbiome of seven different barley genotypes.

	Golden Promise	Morex	HOR7985	BCC1415	BCC436	BCC768	BCC1589
**Proteobacteria**	45.36a	64.87a	48.00a	38.64a	61.60a	50.84a	73.30a
**Actinobacteria**	26.46abc	25.66abc	37.78ab	49.48a	21.30bc	16.91bc	11.16c
**Firmicutes**	23.67a	9.05a	9.48a	10.91a	16.05a	30.81a	15.50a
**Bacteroidetes**	4.12a	0.43b	3.99a	0.55ab	1.03ab	1.03ab	0.02b
**Rare phyla (<1%)**	0.39	0.00	0.76	0.41	0.03	0.41	0.02

Letters indicate significant differences (Tukey's test $p \leq 0.05$).

Table S4. Pielou's evenness, Species richness and Shannon index of bulk soil and barley rhizosphere samples from seven different genotypes grown in mouldboard plough (MP) soil.

	MP Rhizosphere							Bulk soil
	Golden Promise	Morex	HOR7985	BCC1415	BCC436	BCC768	BCC1589	
**Pielou's evenness**	0.96±0.00a	0.95±0.00a	0.95±0.00a	0.96±0.01a	0.95±0.00a	0.95±0.00a	0.95±0.00a	0.95±0.00a
**Species richness**	1625.07±130.97a	868.48±291.52bc	1188.08±188.91ab	659.07±338.83c	1062.38±148.87bc	750.38±74.77bc	979.73±147.30bc	925.32±54.60bc
**Shannon index**	7.07±0.08a	6.41±0.37bc	6.75±0.15ab	6.10±0.51c	6.65±0.13abc	6.31±0.12bc	6.56±0.15abc	6.52±0.07abc

Letters indicate significant differences (Tukey's test $p \leq 0.05$).

Table S5. Pielou’s evenness, Species richness and Shannon index of bulk soil and barley rhizosphere samples from seven different genotypes grown in cultivator tillage (CT) soil.

	CT Rhizosphere							Bulk soil
	Golden Promise	Morex	HOR7985	BCC1415	BCC436	BCC768	BCC1589	
**Pielou’s Evenness**	0.96±0.00a	0.96±0.00a	0.96±0.00a	0.96±0.00a	0.96±0.00a	0.96±0.00a	0.96±0.00a	0.96±0.00a
**Species Richness**	1068.03±133.05ab	904.06±291.92b	1101.27±128.73ab	909.66±88.71b	1170.68±126.99ab	1242.16±244.61ab	1339.95±48.74ab	1496.15±302.94a
**Shannon index**	6.68±0.13ab	6.48±0.37b	6.70±0.11ab	6.51±0.08b	6.75±0.11ab	6.80±0.20ab	6.88±0.04ab	6.99±0.22a

Letters indicate significant differences (Tukey's test $p \leq 0.05$).

Table S6. PERMANOVA of each barley genotype of rhizosphere samples obtained from plants grown in mouldboard plough (MP) soil.

**PERMANOVA MP rhizosphere**	R^2	***p* ≤**
Golden Promise *vs* Morex	0.31	**0.05**
Golden Promise *vs* HOR7985	0.23	**0.05**
Golden Promise *vs* BCC1415	0.37	**0.05**
Golden Promise *vs* BCC436	0.28	**0.05**
Golden Promise *vs* BCC768	0.38	**0.05**
Golden Promise *vs* BCC1589	0.31	**0.05**
Morex *vs* HOR7985	0.18	0.06
Morex *vs* BCC1415	0.15	0.20
Morex *vs* BCC436	0.16	0.11
Morex *vs* BCC768	0.16	0.17
Morex *vs* BCC1589	0.16	0.12
HOR7985 *vs* BCC1415	0.23	**0.05**
HOR7985 *vs* BCC436	0.15	0.23
HOR7985 *vs* BCC768	0.24	**0.05**
HOR7985 *vs* BCC1589	0.19	0.06
BCC1415 *vs* BCC436	0.21	0.06
BCC1415 *vs* BCC768	0.17	0.15
BCC1415 *vs* BCC1589	0.20	**0.05**
BCC436 *vs* BCC768	0.20	**0.05**
BCC436 *vs* BCC1589	0.18	0.06
BCC768 *vs* BCC1589	0.17	**0.05**

Table S7. PERMANOVA of each barley genotype of rhizosphere samples obtained from plants grown in cultivator tillage (CT) soil.

**PERMANOVA CT rhizosphere**	R^2	$p \leq$
Golden Promise *vs* Morex	0.14	0.46
Golden Promise *vs* HOR7985	0.14	0.42
Golden Promise *vs* BCC1415	0.16	0.06
Golden Promise *vs* BCC436	0.16	0.14
Golden Promise *vs* BCC768	0.18	**0.05**
Golden Promise *vs* BCC1589	0.21	**0.05**
Morex *vs* HOR7985	0.14	0.47
Morex *vs* BCC1415	0.14	0.57
Morex *vs* BCC436	0.16	0.18
Morex *vs* BCC768	0.17	0.06
Morex *vs* BCC1589	0.19	**0.05**
HOR7985 *vs* BCC1415	0.15	0.26
HOR7985 *vs* BCC436	0.15	0.17
HOR7985 *vs* BCC768	0.18	**0.05**
HOR7985 *vs* BCC1589	0.18	**0.05**
BCC1415 *vs* BCC436	0.18	0.06
BCC1415 *vs* BCC768	0.18	0.06
BCC1415 *vs* BCC1589	0.22	**0.05**
BCC436 *vs* BCC768	0.17	**0.05**
BCC436 *vs* BCC1589	0.18	**0.05**
BCC768 *vs* BCC1589	0.15	0.13

Table S8. Dominant phyla in bulk soil and barley rhizosphere of seven different genotypes grown in two diverse soils (MP; CT).

	MP								CT							
	**Golden Promise**	**Morex**	**HOR7985**	**BCC1415**	**BCC436**	**BCC768**	**BCC1589**	**Bulk soil**	**Golden Promise**	**Morex**	**HOR7985**	**BCC1415**	**BCC436**	**BCC768**	**BCC1589**	**Bulk soil**
**Acidobacteria**	22.11ab	22.47ab	22.38ab	24.66a	22.62ab	23.13ab	22.58ab	24.34ab	22.52ab	21.97ab	21.21b	22.29ab	22.27ab	23.12ab	22.77ab	22.75ab
**Actinobacteria**	17.75f	20.25cdef	20.09cdef	22.10abcd	21.36bcde	18.14ef	17.27f	23.00abc	19.93cdef	20.03cdef	19.33def	20.58bcdef	18.79def	23.68ab	23.17abc	24.96a
**Proteobacteria**	19.83bcde	21.05abc	18.80cde	18.20def	20.44abcd	22.38ab	22.81a	**15.93f**	20.15abcde	19.36cde	20.36abcd	20.36abcd	20.90abcd	21.43abc	19.91bcde	**17.46ef**
**Planctomycetes**	10.54a	8.64abcd	9.39abc	6.73d	7.96bcd	7.63cd	9.26abc	7.71cd	10.21a	10.23a	9.99ab	8.79abcd	10.00ab	8.86abcd	8.46abcd	8.78abcd
**Chloroflexi**	5.82bc	5.59c	6.21bc	6.10bc	5.70c	6.23bc	6.07bc	**8.49a**	7.23abc	7.17abc	7.40ab	6.91abc	7.15abc	5.86bc	6.54bc	**8.56a**
**Thaumarchaeota**	5.54cde	6.33abcd	7.50ab	7.56ab	5.97abcde	5.86bcde	4.85de	7.72a	5.48cde	6.74abc	6.19abcde	6.70abc	5.78bcde	4.45e	6.09abcde	6.46abcd
**Verrucomicrobia**	7.36a	6.62a	6.20abc	6.68a	5.94abc	6.30ab	7.09a	**3.26d**	6.15abc	6.13abc	6.37a	5.95abc	6.08abc	4.86c	4.93bc	**3.12d**
**Bacteroidetes**	4.05a	3.24abc	3.18abcd	2.06e	3.32ab	2.82bcde	3.55ab	**2.31de**	3.64ab	3.15abcd	3.88a	3.52ab	3.78a	2.34de	2.76bcde	**2.34cde**
**Rare phyla (<2.5%)**	7.01	5.81	6.25	5.91	6.69	7.51	6.51	7.24	4.69	5.22	5.26	4.91	5.26	5.40	5.38	5.57

Letters indicate significant differences (Tukey's test $p \leq 0.05$).

Table S9. Twenty most abundant genera in bulk soil and barley rhizosphere of seven different genotypes grown in two diverse soils (MP; CT).

		MP								CT							
**Phylum**	**Genus**	**Golden Promise**	**Morex**	**HOR7985**	**BCC1415**	**BCC436**	**BCC768**	**BCC1589**	**Bulk soil**	**Golden Promise**	**Morex**	**HOR7985**	**BCC1415**	**BCC436**	**BCC768**	**BCC1589**	**Bulk soil**
**Acidobacteria**	**Uncl. *Subgroup 6***	11.73	12.19	13.18	14.65	12.82	13.21	12.36	14.16	13.85	13.47	13.21	13.84	13.60	14.80	14.34	14.39
**Thaumarchaeota**	**Uncl. *Nitrososphaeraceae***	4.95	5.85	6.77	7.13	5.44	5.48	4.47	7.04	4.51	5.73	5.13	5.51	4.74	3.83	5.16	5.29
**Planctomycetes**	**Uncl. *WD2101 soil group***	3.45	2.42	2.68	1.71	2.27	2.50	2.87	1.97	2.84	2.79	2.45	2.20	2.54	1.73	1.80	1.91
**Alphaproteobacteria**	***Sphingomonas***	2.39	2.95	2.07	2.26	2.80	2.88	2.50	2.43	1.93	1.55	1.77	1.64	2.22	2.21	2.45	1.71
**Actinobacteria**	**Uncl. *Micrococcaceae***	2.00	3.10	2.42	3.16	2.77	2.97	2.35	1.35	1.57	1.86	1.65	1.71	1.91	2.35	2.20	1.29
**Gammaproteobacteria**	**Uncl. *Burkholderiaceae***	2.25	2.25	2.07	2.13	2.07	2.91	2.60	1.45	2.30	2.01	2.00	2.31	2.46	2.02	1.75	1.40
**Acidobacteria**	**Uncl. *Blastocatellaceae***	2.60	2.73	1.96	2.86	2.35	2.24	2.24	2.06	1.72	1.68	1.64	1.75	1.79	1.71	1.59	1.47
**Chloroflexi**	**Uncl. *KD4-96***	1.50	1.65	1.69	2.06	1.88	1.74	1.65	2.40	1.91	1.90	2.06	2.11	2.02	2.24	2.11	2.91
**Actinobacteria**	***Gaiella***	1.35	1.57	1.77	1.95	1.80	1.48	1.40	2.33	2.04	2.25	1.82	2.10	1.80	2.15	2.16	2.46
**Acidobacteria**	***RB41***	2.56	2.58	2.29	2.43	2.16	2.48	2.46	3.04	1.29	1.38	1.30	1.33	1.33	1.13	1.17	1.37
**Gammaproteobacteria**	***Massilia***	1.09	1.95	1.41	1.59	1.76	2.43	3.01	0.27	2.47	1.88	2.49	2.77	2.60	2.21	1.88	0.24
**Actinobacteria**	***Nocardioides***	1.08	1.63	1.43	2.00	1.48	1.35	1.32	1.78	1.96	1.98	1.78	1.95	1.59	2.10	2.19	2.22
**Verrucomicrobia**	***Chthoniobacter***	2.30	2.19	1.91	1.92	2.10	1.69	2.07	0.68	1.83	1.82	1.94	1.71	1.98	1.35	1.31	0.70
**Actinobacteria**	**Uncl. *Gaiellales***	1.56	1.72	1.90	1.69	1.94	1.27	1.38	2.50	1.52	1.25	1.50	1.42	1.39	1.66	1.78	2.29
**Actinobacteria**	**Uncl. *MB-A2-108***	1.61	1.85	2.04	2.00	2.00	1.79	1.97	2.44	1.44	1.50	1.30	1.44	1.18	1.36	1.37	1.49
**Verrucomicrobia**	***Luteolibacter***	2.09	1.61	1.43	2.08	1.31	1.98	2.07	0.12	1.76	1.61	1.73	1.57	1.33	1.20	1.23	0.22
**Planctomycetes**	***Pirellula***	1.49	1.31	1.32	1.01	1.13	0.96	1.21	0.81	1.52	1.29	1.67	1.41	1.45	1.69	1.36	1.17
**Gemmatimonadetes**	**Uncl. *Gemmatimonadaceae***	1.4	1.08	1.15	0.83	1.09	1.49	1.42	1.97	1.29	1.29	1.34	1.22	1.29	0.85	1.16	1.46
**Planctomycetes**	**Uncl. *Pirellulaceae***	1.31	1.15	1.30	1.07	1.09	0.68	1.17	1.07	1.19	1.11	1.07	1.14	1.17	1.12	0.98	1.30
**Actinobacteria**	***Streptomyces***	1.02	1.03	1.11	1.07	1.24	1.08	0.88	1.17	0.88	0.86	0.92	0.92	0.90	1.26	1.17	1.32

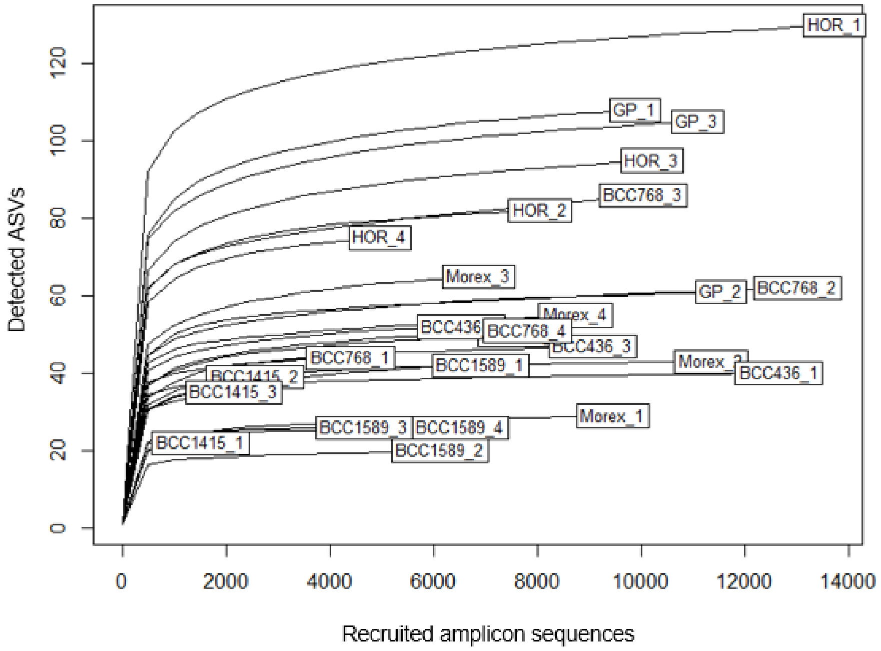

Figure S1. Rarefaction curves of 16S rRNA gene amplicon sequencing data of the barley seed microbiome.

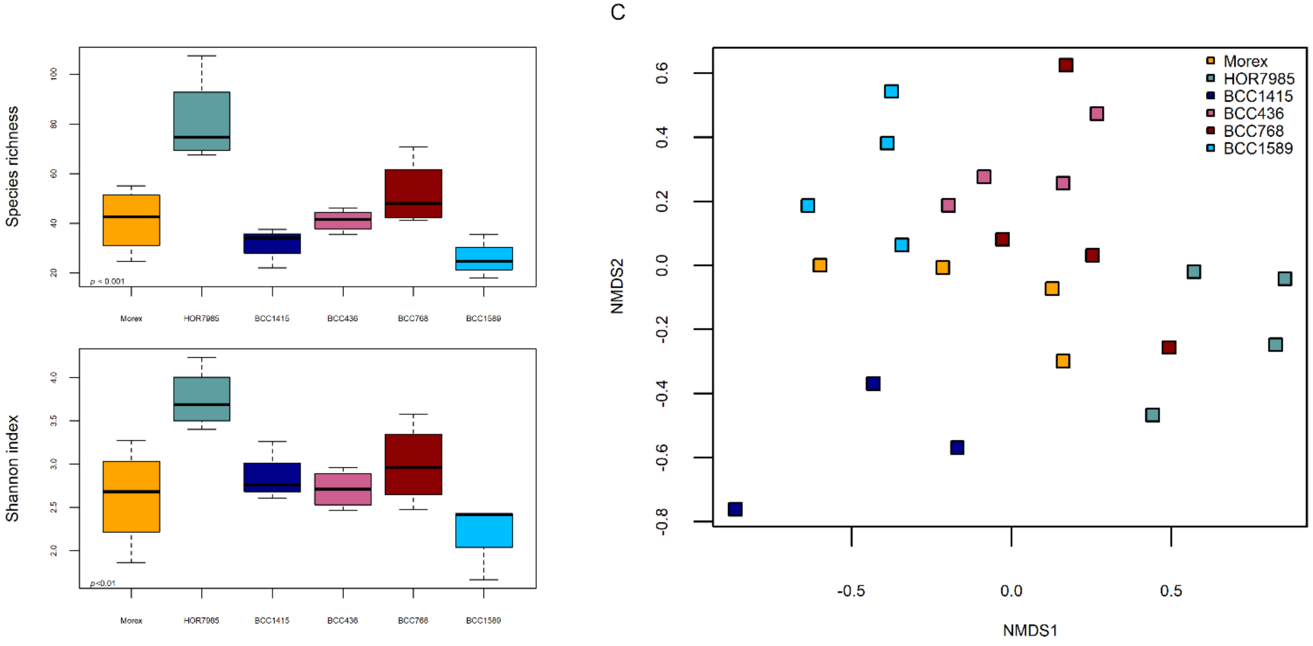

Figure S2 Microbial diversity of the endophytic seed microbiome varied between six barley genotypes originated from the same place. The microbial alpha-diversity indices Species richness (A) and Shannon diversity index (B) varied depending on the plant genotype (ANOVA for Species richness $p \leq 0.001$ and Shannon index $p \leq 0.01$). Beta-diversity of the endophytic seed microbiome was visualized by NMDS (C). The microbiome composition based on Bray-Curtis community dissimilarities (ASVs obtained from 16S rRNA gene amplicon sequencing) and was assessed from DNA of surface-sterilized barley seeds of six genotypes harvested at the same field site.

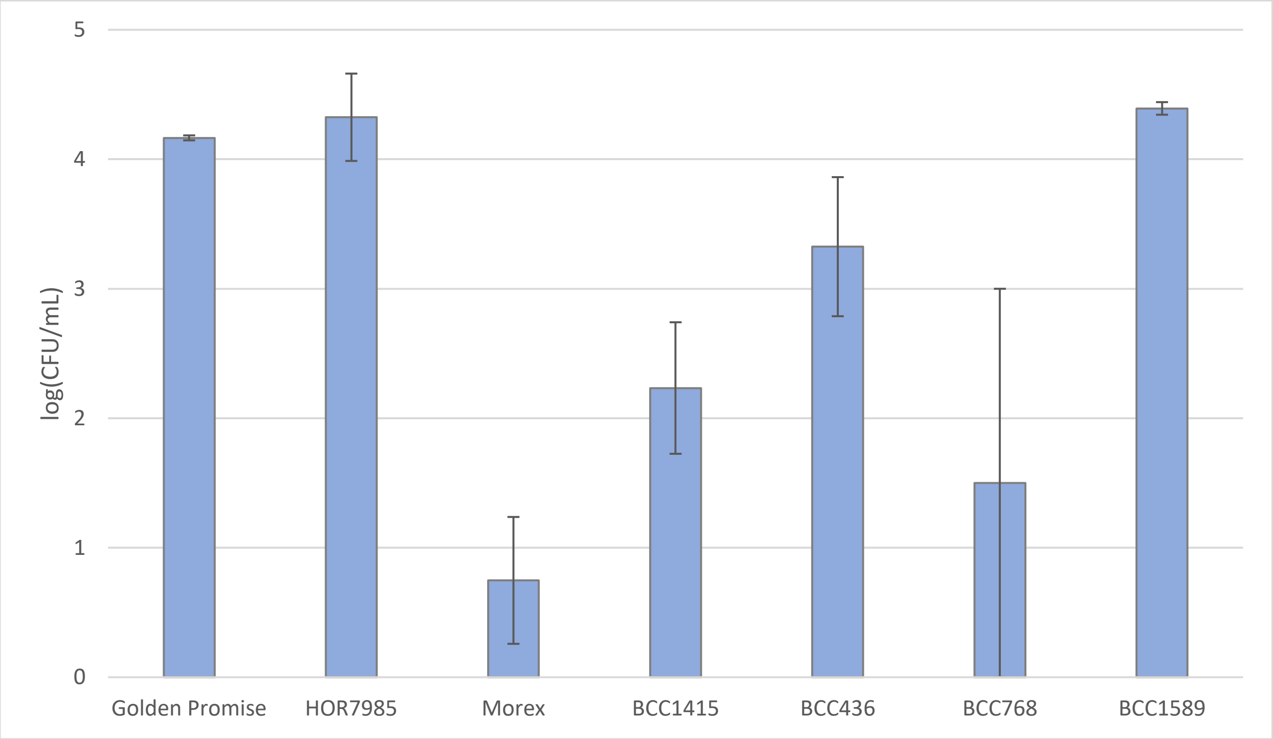

Figure S3. Logarithm of colony forming units (CFU)/g seed of seven different barley genotypes determined after 48 h incubation at 28°C. 0.5 g of surface-sterilized seeds were grounded and solved in 4.5 mL sterile double-distilled water. The seed suspension was plated on R2A supplemented with 100 µg/mL cycloheximide.

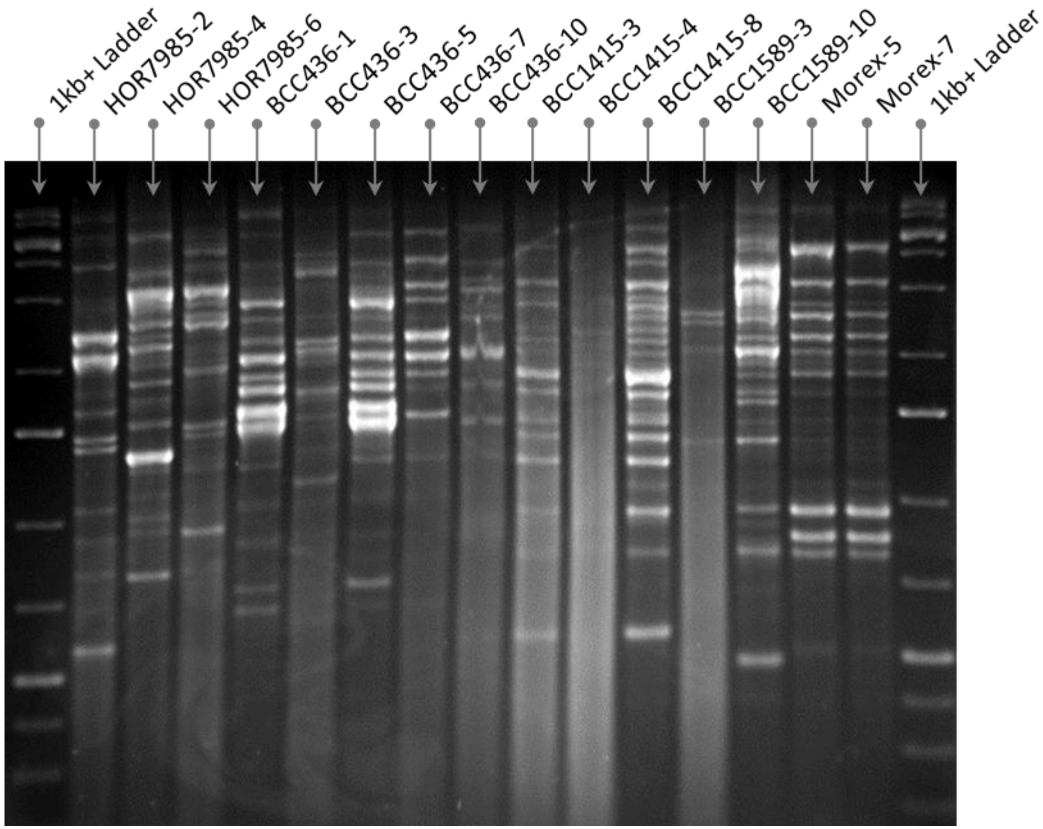

Figure S4. BOX fingerprint of isolated endophytes taxonomically affiliated to the genus *Curtobacterium*.

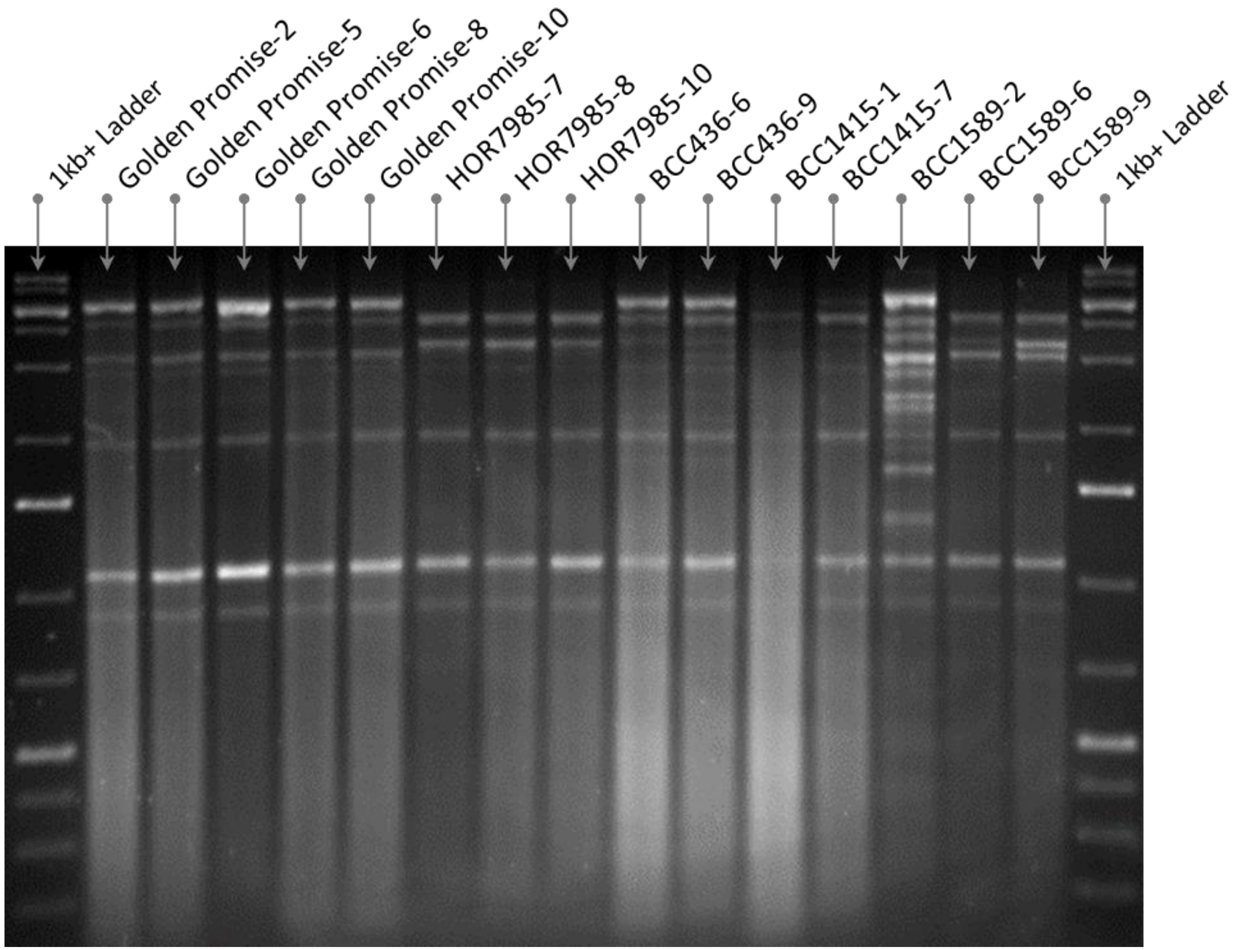

Figure S5. BOX fingerprint of isolated endophytes taxonomically affiliated to the genus *Paenibacillus.*

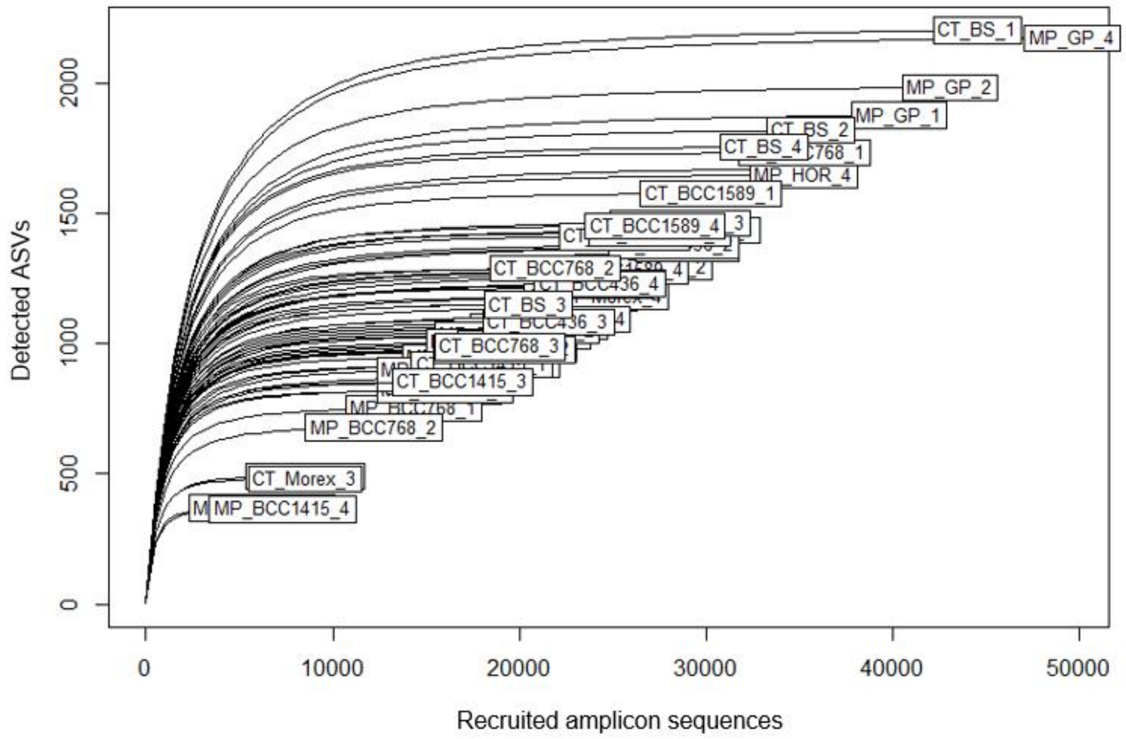

Figure S6. Rarefaction curves of 16S rRNA gene amplicon sequencing data of barley rhizosphere and bulk soil samples from two different soils (MP: mouldboard plough; CT: cultivator tillage) and seven different genotypes.

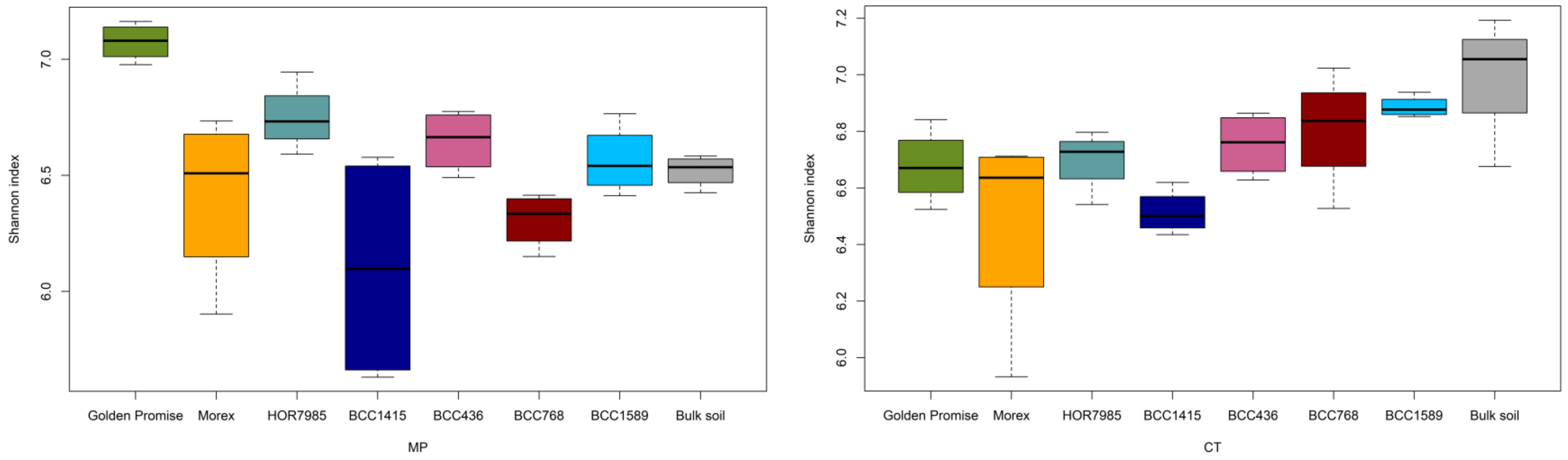

Figure S7. Shannon index of bulk soil and barley rhizosphere samples of seven different genotypes grown in MP and CT soils.

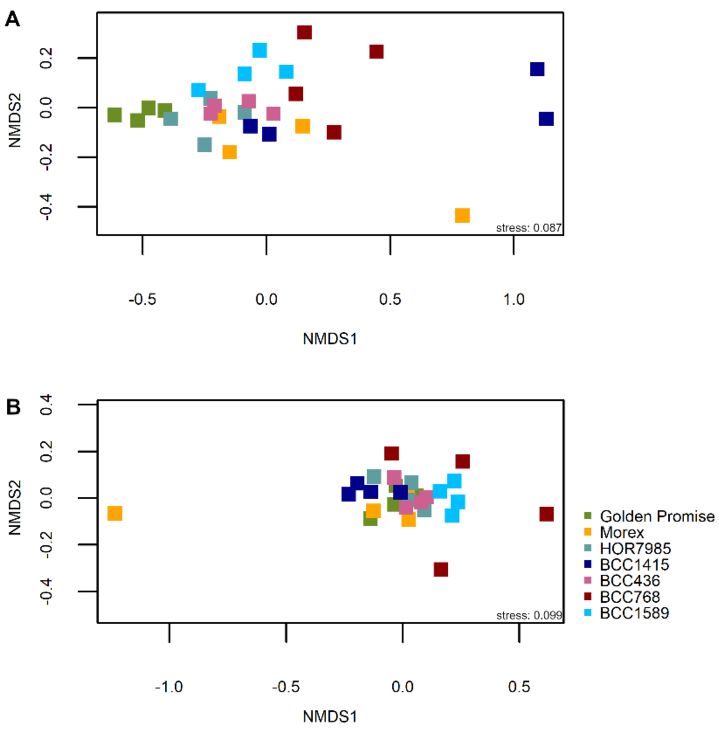

Figure S8. Non-metric multidimensional scaling (NMDS) of barley rhizosphere microbial communities from seven different genotypes grown in MP (A) and CT (B) soil. The rhizosphere community composition based on Bray-Curtis dissimilarities and was obtained from ASVs. The seven different genotypes were grown in MP (mouldboard plough) and CT (cultivator tillage) soil until BBCH13. Respective bulk soil samples are not shown. ANOSIM verified significant differences between the genotypes ($p \leq 0.001$).
